# Prosaposin and its receptors GRP37 and GPR37L1 show increased immunoreactivity in the facial nucleus following facial nerve transection

**DOI:** 10.1371/journal.pone.0241315

**Published:** 2020-12-01

**Authors:** Joji Kunihiro, Hiroaki Nabeka, Hiroyuki Wakisaka, Kana Unuma, Md. Sakirul Islam Khan, Tetsuya Shimokawa, Farzana Islam, Takuya Doihara, Kimiko Yamamiya, Shouichiro Saito, Fumihiko Hamada, Seiji Matsuda

**Affiliations:** 1 Department of Anatomy and Embryology, Ehime University Graduate School of Medicine, Toon, Ehime, Japan; 2 Department of Otorhinolaryngology, Ehime University Graduate School of Medicine, Toon, Ehime, Japan; 3 Section of Forensic Medicine, Graduate School of Medical and Dental Sciences, Tokyo Medical and Dental University, Bunkyo, Tokyo, Japan; 4 Laboratory of Veterinary Anatomy, Faculty of Applied Biological Sciences, Gifu University, Yanagido, Gifu, Japan; 5 Department of Human Anatomy, Oita University Faculty of Medicine, Yufu, Oita, Japan; Martin Luther University, GERMANY

## Abstract

Neurotrophic factor prosaposin (PS) is a precursor for saposins A, B, C, and D, which are activators for specific sphingolipid hydrolases in lysosomes. Both saposins and PS are widely contained in various tissues. The brain, skeletal muscle, and heart cells predominantly contain unprocessed PS rather than saposins. PS and PS-derived peptides stimulate neuritogenesis and increase choline acetyltransferase activity in neuroblastoma cells and prevent programmed cell death in neurons. We previously detected increases in PS immunoactivity and its mRNA in the rat facial nucleus following facial nerve transection. PS mRNA expression increased not only in facial motoneurons, but also in microglia during facial nerve regeneration. In the present study, we examined the changes in immunoreactivity of the PS receptors GPR37 and GPR37L1 in the rat facial nucleus following facial nerve transection. Following facial nerve transection, many small Iba1- and glial fibrillary acidic protein (GFAP)-positive cells with strong GPR37L1 immunoreactivity, including microglia and astrocytes, were observed predominately on the operated side. These results indicate that GPR37 mainly works in neurons, whereas GPR37L1 is predominant in microglia or astrocytes, and suggest that increased PS in damaged neurons stimulates microglia or astrocytes via PS receptor GPR37L1 to produce neurotrophic factors for neuronal recovery.

## 1 Introduction

Prosaposin (PS) is the precursor protein of four small lysosomal glycoproteins called saposins A, B, C, and D [1]. PS is transported into the lysosome, and there it is proteolytically processed into the four saposins, which are necessary for the normal hydrolysis of sphingolipids in the lysosome [2,3]. Both saposins and PS are widely expressed in various tissues, although the brain, skeletal muscle, and heart cells predominantly contain unprocessed PS rather than saposins [4]. Various secretary fluids, such as cerebrospinal fluid, human breast milk, seminal plasma, bile, and pancreatic juice, contain unprocessed prosaposin (PS) [5,6]. Hence, PS may have pivotal functions other than serving as a precursor for saposins.

PS has been identified as a potent neurotrophic factor [7]. PS and peptides containing the neurotrophic activity domain of PS, promoted neurite outgrowth, elevated choline acetyltransferase activity in neuroblastoma cells [7], and prevented programmed cell death of cerebral granule neurons *in vitro* [8]. In *in vivo* experiments, PS and PS-derived peptides facilitated sciatic nerve regeneration after transection [9] and rescued hippocampal CA1 neurons after brain ischemia [10,11]. The PS-derived peptide also shows potent neurotrophic activity in ischemia-induced hearing loss [12], kainic acid-induced brain injury [13–15], and amyloid-beta neurotoxicity [16]. As well as neurotrophic activity, PS shows strong gliotrophic activity that prevents cell death and increases myelin constituents in cultured Schwann cells and oligodendrocytes [17,18].

GPR37 and GPR37L1, orphan G-protein-coupled receptors (GPCRs), were recently defined as PS receptors [19]. Both receptors are widely expressed in the brain, where GPR37 is linked to Parkinson’s disease [20], and GPR37L1 deletion leads to precocious cerebellar development [21]. Glioprotection and neuroprotection by prosaposin (PS) have been reported to be mediated by GPR37 and GPR37L1 [22,23]; however, the specific associations between PS and these receptors are not yet universally acknowledged (reviewed in Smith [24]).

Previously, using specific antibodies against PS and these receptors, we showed that PS expression in Purkinje cells and interneurons in the cerebellum was markedly enhanced following kainic acid treatment [15]. We also reported that the two PS receptors show roughly complementary distributions in the rat testis; GPR37L1 is upregulated in germ cells in the earlier stages of spermatogenesis, whereas GPR37 becomes predominant in the later stages [25].

Previously, an increase in PS immunoreactivity [26] and its mRNA expression [27] was detected in rat facial nuclei following facial nerve transection. Meyer et al. [19] predicted that “Future work may determine the precise roles of GPR37 and GPR37L1 with the *in vivo* model which strikingly up-regulate PS following nerve injury [27]”. Therefore, we examined the expression patterns of PS receptors in the same *in vivo* model. In the present study, we observed many microglial cells and some astrocytes with GPR37L1 immunoreactivity on the operated side after facial nerve transection.

## 2 Materials and methods

### 2.1 Animals

Male Wistar rats (8 weeks old, 200–250 g body weight) were used in this study. All animals were provided by SLC Japan (Shizuoka) and were housed at 22°C under a 12:12 h light/dark cycle with food and water provided ad libitum. The experiments were conducted in accordance with ARRIVE guidelines and the guidelines for animal experimentation at Ehime University School of Medicine, Japan. The protocol was approved by the Animal Care Committee of Ehime University (Permit Number: 05A261). Surgical operations were carried out as previously reported [26,27].

### 2.2 Antibodies

The anti-rat PS-specific antibody (IM-1) was prepared by Medical and Biological Laboratories (Nakaku, Nagoya, Japan). From the amino acid sequence of rat PS, a synthetic oligopeptide corresponding to the proteolytic portion (IM: the intermediate portion between saposins C and D) of PS (aa 409–434) was used to generate a rabbit polyclonal antibody against rat PS. The sequence did not encode any saposins, but was obtained from a PS amino acid sequence analysis [28]. Therefore, IM-1 does not react to any saposins, but reacts to PS, trisaposin (saposin B-C-D), and disaposin (saposin C-D).

Specific polyclonal antibodies against two receptors were generated by Eurofins Genomics (Tokyo, Japan), and all procedures were performed as previously described [28–30]. Briefly, specific antibodies were created by immunizing rabbits with synthetic oligopeptides based on rat amino acid protein sequences specific to PS: M19936 [31,32], GPR37 (NP 476549.1) [33], or GPR37L1 (NP 665727.2) [34]. The sequences used were:

PS(IM-1): 409-PKEPAPPKQPEEPKQSALRAHVPPQK-434;
GPR37: 134-REPTDSQLFRQTSE-147 (#12795V);
and GPR37L1: 286-CIMKPSADLPESLYS-300 (#12796V).

To identify neurons, monoclonal anti-neuronal nuclei antibody (NeuN, Chemicon, Temecula, CA, USA) was used. Three antibodies, polyclonal goat anti-Iba1 (Abcam, Tokyo, Japan), monoclonal anti-glial fibrillary acidic protein (GFAP, Sigma, St. Louis, MO, USA), and monoclonal anti-O4 antibody (Chemicon, Temecula, CA, USA), were used to identify glial cells for the tree glial subtypes, namely microglia, astrocytes, oligodendrocytes, respectively.

### 2.3 Surgical procedures and tissue preparation

**Fig 1:**
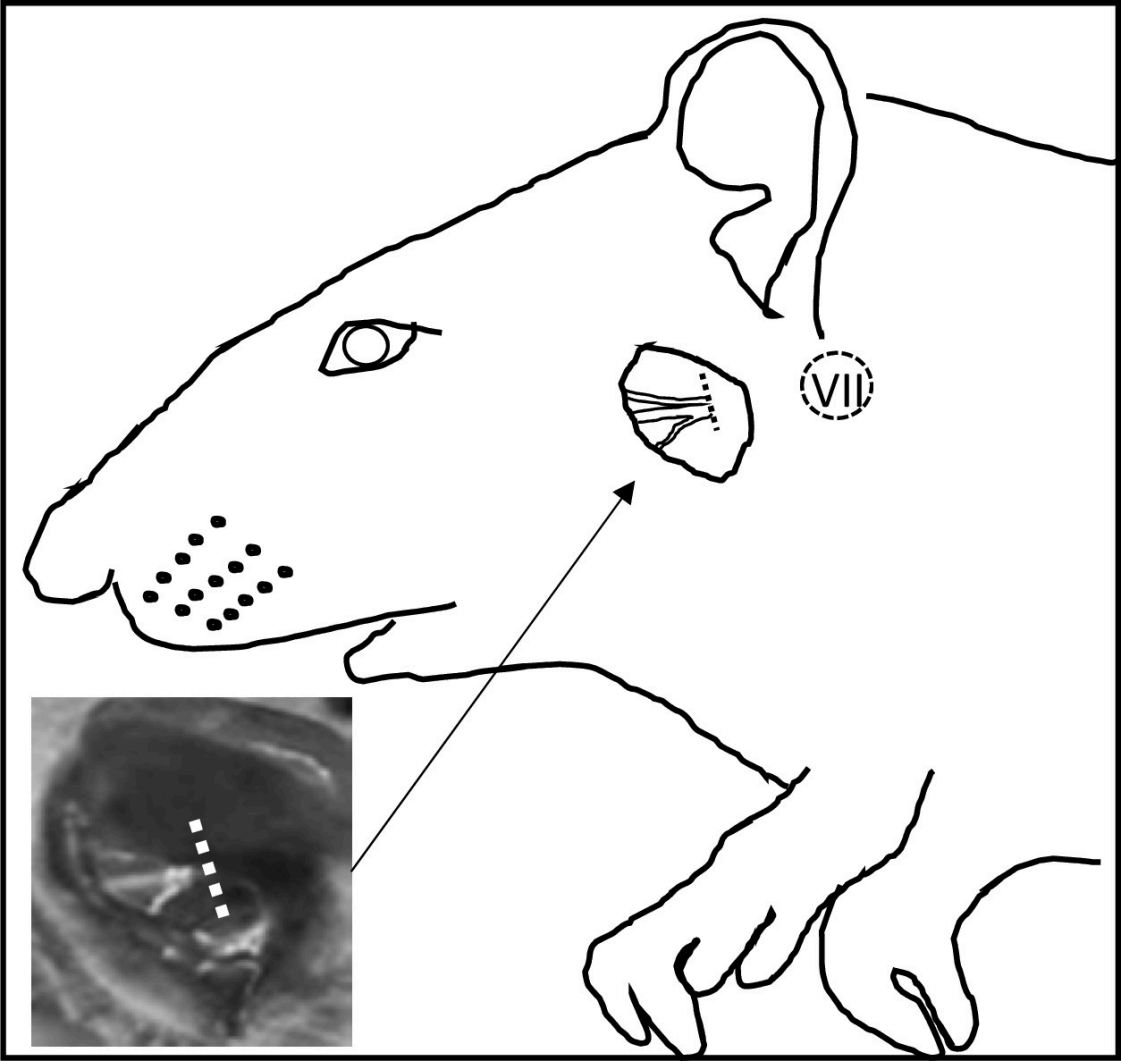
Drawing based on a photograph of an operated rat. The inset photograph shows only the operated portion. The left facial nerve at the level of the stylomastoid foramen was transected. The right side was untreated. The dotted circle indicates the estimated portion of the facial nucleus.

The following experiments were conducted in accordance with the guidelines for animal experimentation at Ehime University School of Medicine, Japan. Surgical operations were carried out as reported previously [26]. After male Wistar rats (8 weeks old, 200–250 g body weight) were anesthetized by intraperitoneal injection of medetomidine (0.3 mg/kg), midazolam (4 mg/kg) and butorphanol (5 mg/kg). After anesthesia, the left facial nerve was transected at the level of the stylomastoid foramen (Fig 1). The facial nerves were not sutured. The right side was untreated.

We anesthetized eight animals on postoperative day 7, and four animals each on postoperative days 1 and 3. After anesthesia, animals were euthanized by cardiac perfusion with physiological saline, and fixed in 4% paraformaldehyde and 0.1 M phosphate-buffered saline (PBS, pH 7.4). After fixation, the brain stems were dissected, post-fixed in the same solution for 4 h, and then embedded in paraffin using conventional methods, sectioned, and deparaffinized.

### 2.4 Immunohistochemistry of PS, GPR37, and GPR37L1

For the immunohistochemistry and immunofluorescence of GPR37 and GPR37L1, boiling for antigen retrieval is necessary, but it is not necessary for PS. Following deparaffinization, brain sections were boiled for 20 min and cooled in 10 mM sodium citrate, after which the sections were exposed for 2 h to a blocking solution containing 1% normal goat serum, 5% bovine serum albumin, 0.2% fish gelatin, 0.1% triton X, and 0.1% NaN_3_ in PBS. The sections were processed for immunohistochemistry with anti-PS (IM-1), anti-GPR37, and anti-GPR37L1 at a concentration of 1 μg/mL overnight at 4°C. The sections were then rinsed with PBS and incubated with biotinylated anti-rabbit IgG (Dako) for 2 h at 32°C. After rinsing again with PBS, the sections were incubated for 30 min at 32°C with peroxidase-conjugated streptavidin (Vector). The sections were rinsed with PBS and subjected to a color reaction with diaminobenzidine (DAB).

### 2.5 Immunofluorescence

The sections were processed for immunofluorescence by incubating with rabbit IgG against GPR37 or GPR37L1, and mouse IgG against GFAP, Iba-1, O4, or NeuN at 1 μL/mL two overnight at 4°C. After washing with PBS, the sections were treated for 2 h at room temperature with Alexa Fluor 594-conjugated goat-anti-rabbit IgG (1:500; Rockland, Gilbertsville, PA, USA) for detection of GPR37 or GPR37L1; with Alexa Fluor 488-conjugated goat-anti-mouse IgG (1:500; Rockland, Gilbertsville, PA, USA) for detection of GFAP, O4 or NeuN; with Alexa Fluor 488-conjugated sheep anti-goat IgG (1:500; Rockland, Gilbertsville, PA, USA) for detection of Iba1; and with 4’,6-diamidino-2-phenylindole (DAPI, 1:1000) (Figs 3–5). After washing with PBS, the sections were mounted in Mowiol (Calbiochem, San Diego, CA, USA), and examined under a Nikon A1 confocal microscope (Nikon, Tokyo, Japan).

### 2.6 Three-dimensional laser scanning confocal analysis

The immunofluorescence images were reconstructed with a computer, using three-dimensional imaging software (NIS-Elements 4.50, Nikon, Tokyo, Japan). Image acquisition was performed in z-scan automatic volume mode and a series of 30 images 129 × 129 μm to a depth of 10 μm were acquired, from which the three-dimensional renderings were obtained.

### 2.7 Statistical analysis

**Fig 2:**
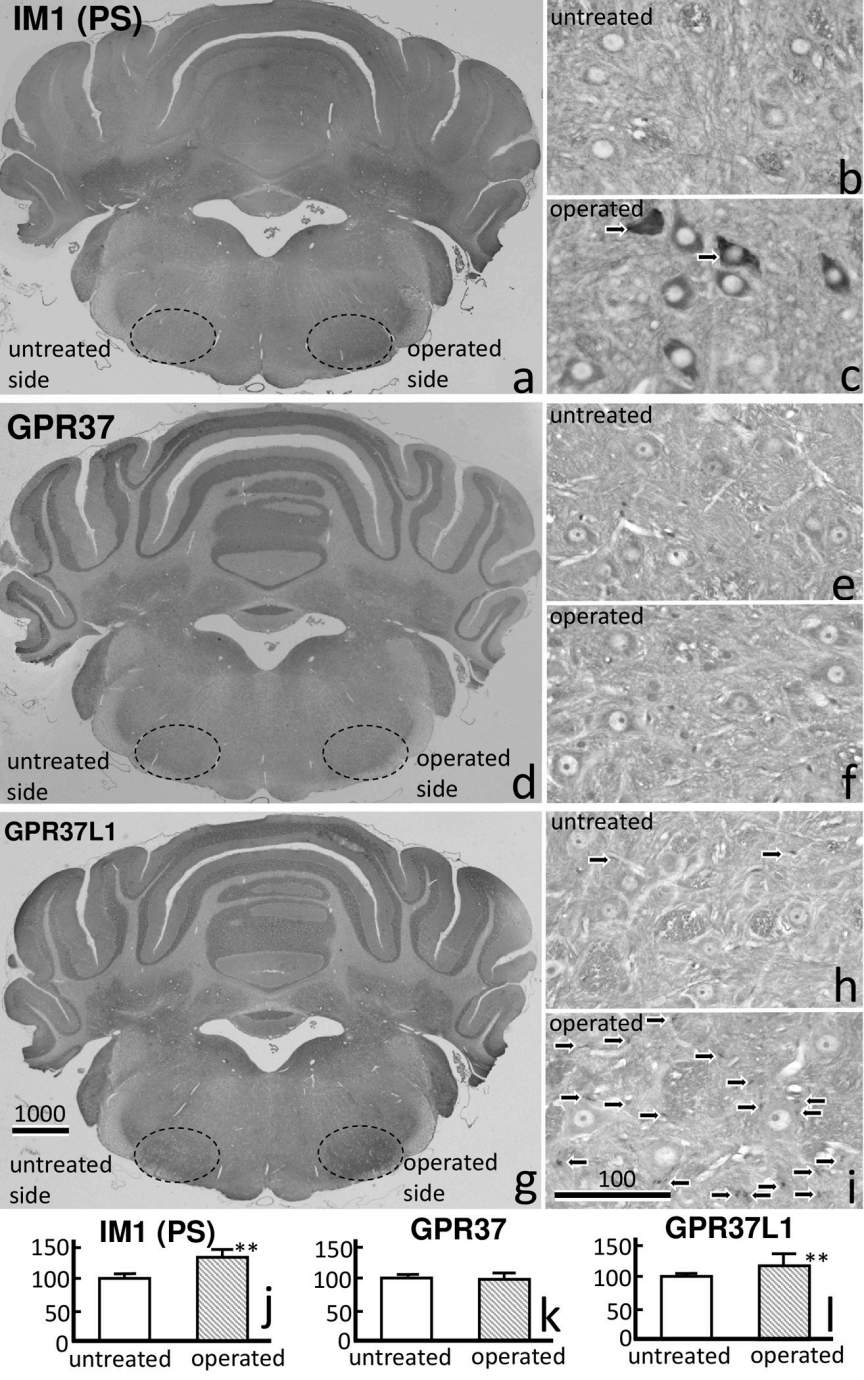
Prosaposin (PS:IM1), GPR37, and GPR37L1 immunoreactivity (IR) with DAB staining in facial nucleus 7 days after transection of the facial nerve. A) PS-IR clearly increased on the operated side. PS-IR was mainly observed in the motoneurons on the operated side (c) compared with those on the untreated side (b). d) GPR37-IR showed a weak increase on the operated side. e, f) A weak GPR37-IR signal was observed in the motoneurons on both sides. g) A strong GPR37L1-IR signal was observed around the motoneurons, predominately on the operated side. GPR37L1-IR was observed predominately in the glial cells (arrows) around the neurons on the operated side (i), and rarely on the untreated side (h). The numbers on the bars indicate the scale in microns. (j) PS-IR and (l) GPR37L1-IR were significantly higher on the operated side than on the untreated side (p** < 0.01).

We analyzed the relative PS-IR intensity (Fig 2) and GPR37L1-positive glial cell numbers using a one-way analysis of variance (ANOVA) and paired *t*-tests. All statistical analyses were performed using StatView software (Abacus Concepts Inc., Berkeley, CA, USA).

## 3 Results

### 3.1 Immunohistochemistry of PS, GPR37, and GPR37L1

Immunoreactivity (IR) of PS, GPR37, and GPR37L1 in the facial nuclei on postoperative day 7 was compared between the operated and untreated sides (Fig 2). A large increase in PS-IR on the operated side was evident even at low magnification (Fig 2A). At higher magnification, increased PS-IR was mainly observed in the facial motoneurons, predominately on the operated side (Fig 2C) compared with the untreated side (Fig 2B).

A weak increase in GPR37-IR on the operated side was observed on postoperative day 7 (Fig 2D). At high magnification, GPR37-IR was observed in the facial nuclei on both sides (Fig 2E and 2F). GPR37-IR in the cytoplasm of the motoneurons was more intense around the cell nuclei than in the periphery (Fig 2E and 2F). GPR37 L1-IR also increased on the operated side, even at low magnification (Fig 2G). At higher magnification, GPR37L1-IR was predominantly observed in the facial nucleus on the operated side (Fig 2I) compared with the untreated side, where almost no GPR37L1-IR was observed (Fig 2H). It appears that the GPR37L1-IR cells were glial cells surrounding the neurons (Fig 2I). In the present study, we observed large numbers of microglial cells and some astrocytes with GPR37L1 immunoreactivity (IR) on the operated side following facial nerve transection. The IR data for PS-IR, GPR37, and GPR37L1 are shown in Fig 2. PS-IR and GPR37L1-IR were significantly higher on the operated side than on the untreated side.

**Fig 3:**
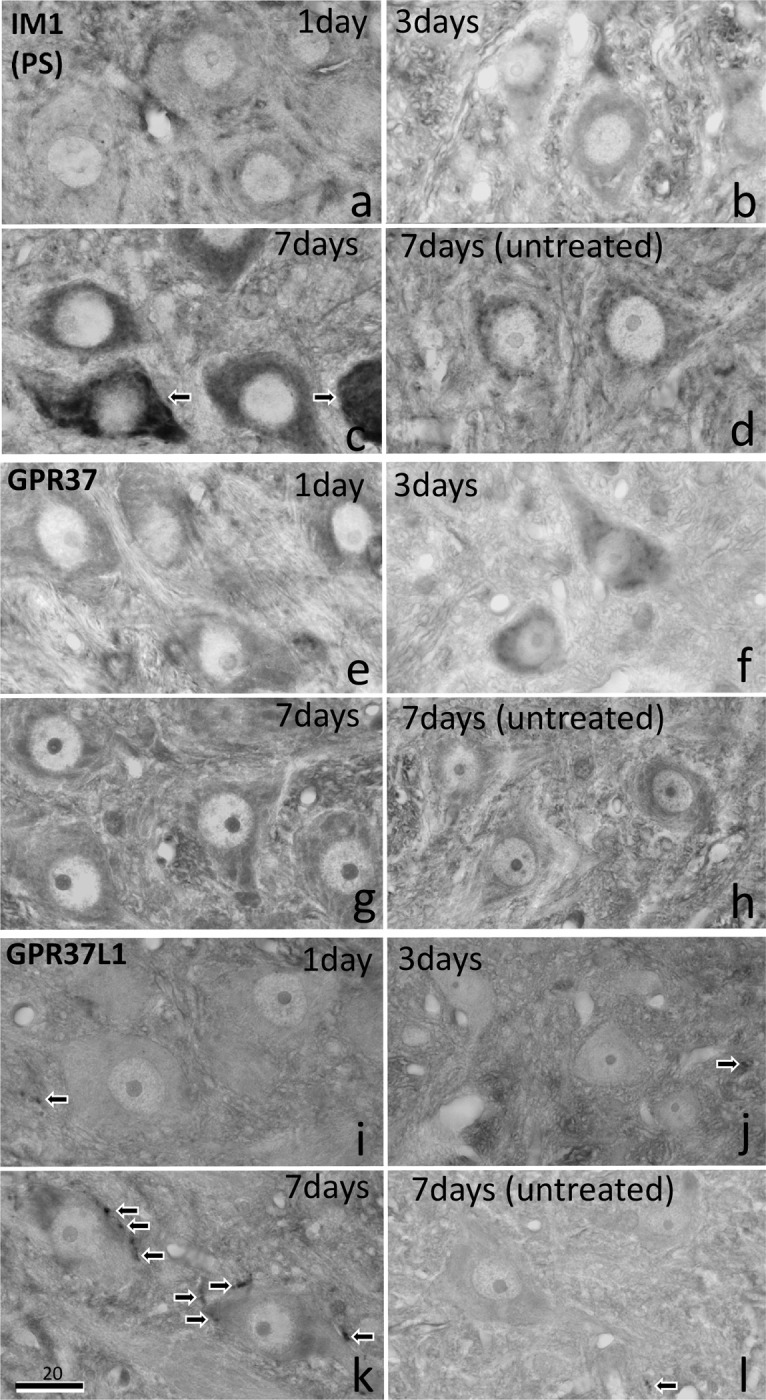
PS, GPR37, and GPR37L1 immunoreactivity (IR) with DAB staining on days 1, 3, and 7 after facial nerve transection. PS-IR decreased slightly on day 1 (a), but increased moderately on day 3, and increased strongly on day 7 (c). Some motoneurons showed very intense PS-IR signal (arrows), whereas others were moderate. GPR37-IR showed a weak increase in the motoneurons on the operated side on days 1, 3, and 7 (e, f, g) compared with the levels on the untreated side (h). GPR37 L1-IR signal was observed in glial cells (arrows) around the motoneurons of the facial nucleus predominately on the operated side on day 7 (k), but not on the untreated side (l) or on days 1 or 3 (i, j). The numbers on the bars indicate the scale in microns.

Variation in PS-IR over time is shown in Fig 3 for postoperative days 1 (n = 4), 3 (n = 4), and 7 (n = 8). PS-IR on the operated side was slightly decreased on day 1 (Fig 3A) compared with that on the untreated side (Fig 3D), moderately increased on day 3, and greatly increased on day 7 (Fig 3B and 3C). GPR37-IR showed weak increases on days 1, 3, and 7 on the operated side (Fig 3E-3G) compared with that on the untreated side (Fig 3H). A strong increase in GPR37L1-IR was observed on day 7 on the operated side (Fig 3K), but not on days 1 or 3 (Fig 3I and 3J) or on the untreated side (Fig 3L).

### 3.2 Immunofluorescence

**Fig 4:**
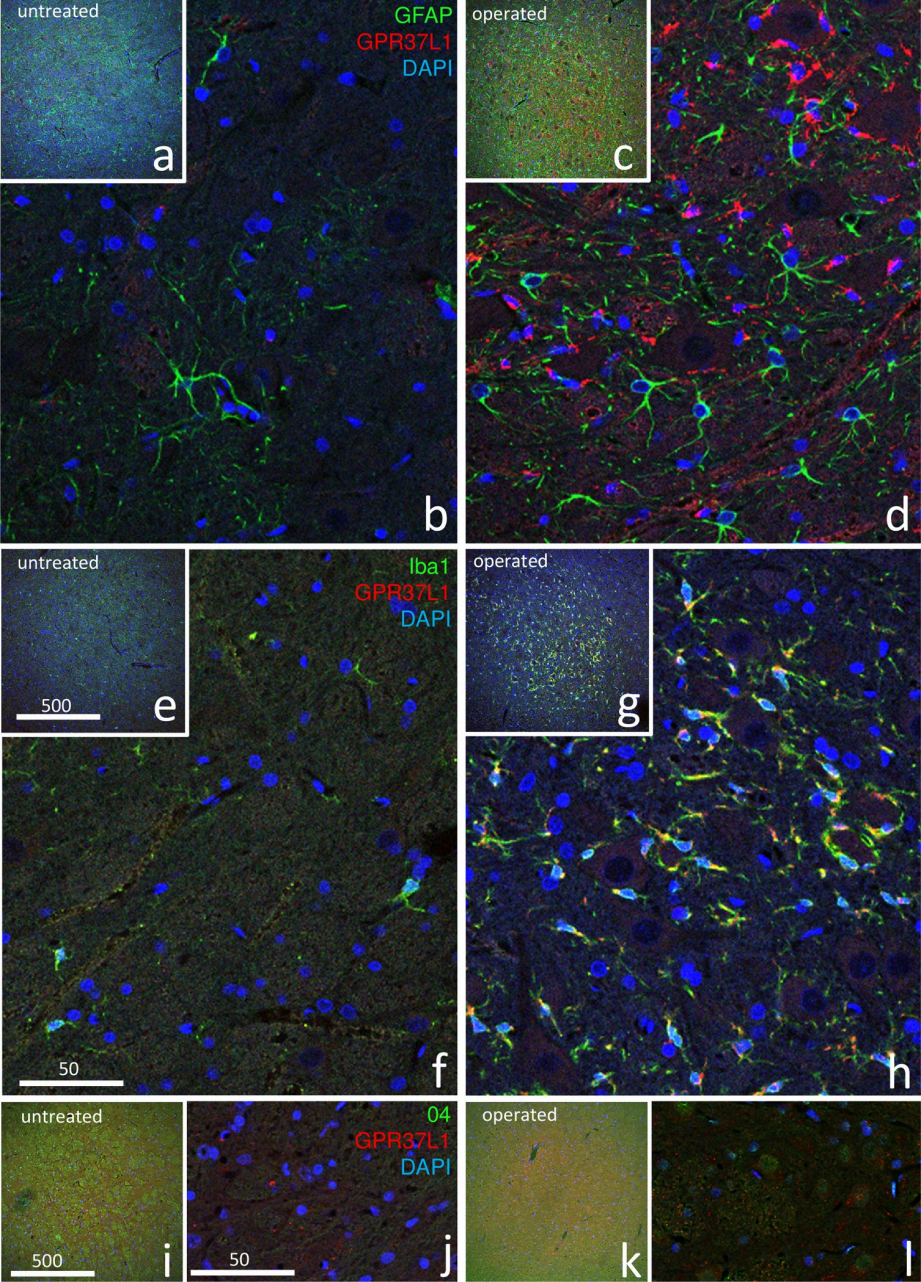
Double-immunofluorescence staining in the facial nucleus 7 days after facial nerve transection. Double-staining images for GPR37L1 (red) with glial markers (GFAP, Iba1, and O4; green) and DAPI (blue) are shown at lower (a, c, e, g, i, k) and higher (b, d, f, h, j, l) magnification. These images show that cells with a high GPR37L1-IR signal were located predominately on the operated side (d, h, l). Double-staining with GPR37L1 and GFAP (a–d) showed that GPR37L1-IR and GFAP-IR increased intensely in the facial nerves on the operated side (c, d), but co-localization of GPR37L1 with GFAP was not evident (d). Double-staining with GPR37L1 and Iba1 (e–h) showed that GPR37L1-IR and Iba1-IR also increased on the operated side (g, h), and high co-localization of GPR37L1 with Iba1 was observed in many cells (h). Double-staining with GPR37L1 and O4 (i–l) showed that GPR37L1-IR increased, but O4-IR cells only increased weakly on the operated side (k, l), and co-localization of GPR37L1 with O4 was not evident (l). The numbers on the bars indicate the scale in microns.

Fig 4 shows the results of double-staining with GPR37L1 and glial markers (GFAP, Iba1, O4) at postoperative day 7. All three images show that GPR37L1-IR cells increased predominately on the operated side. Double-staining with GPR37L1 and GFAP (Fig 4A-4D) showed that both GPR37L1-IR and GFAP-IR greatly increased in the facial nucleus on the operated side (Fig 4C and 4D) compared with that on the untreated side (Fig 4A and 4B), but co-localization of GPR37L1 with GFAP was not evident (Fig 4D). Double-staining with GPR37L1 and Iba1 (Fig 4E–4H) showed that both GPR37L1-IR and Iba1-IR greatly increased on the operated side (Fig 4G and 4H), and co-localization of GPR37L1 with Iba1 was observed in many cells (Fig 4H). Double-staining with GPR37L1 and O4 (Fig 4I–4L) showed a strong increase in GPR37L1-IR but only a weak increase in O4-IR on the operated side (Fig 4K and 4L), and co-localization of GPR37L1 with O4 was not evident (Fig 4L).

**Fig 5:**
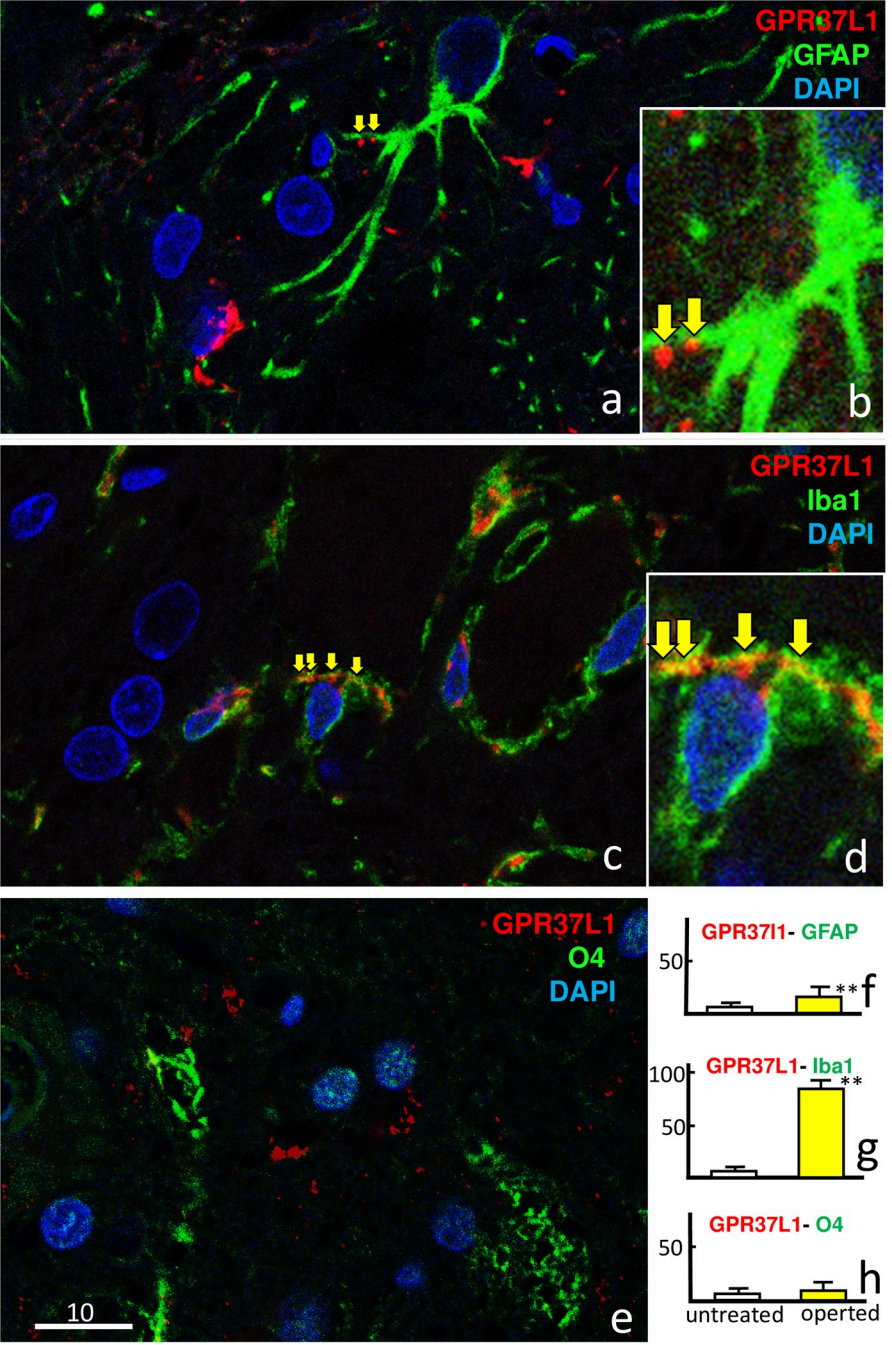
Facial nucleus double-staining with GPR37L1 (red) and glial markers (GFAP, Iba1, and O4; green) and 4′,6-diamidino-2-phenylindole (DAPI; blue) 7 days after facial nerve transection, at different magnifications. Co-localization (yellow arrows) of GPR37L1 with GFAP was not obvious at low magnification (a), but clearly observed at high magnification (b). Co-localization of GPR37L1 with Iba1 was frequently observed even at low magnification (c, d). Co-localization of GPR37L1 with O4 was not obvious (e). The numbers of double-positive cells per 100,000 μm^2^ were counted at high magnification (f–h). Double-positive GPR37L1 cells stained with (f) GFAP or (g) Iba1 were significantly more frequent on the operated side than on the untreated side (p** < 0.01). The scale bar units are microns.

The results of facial nucleus double-staining with GPR37L1, glial markers (GFAP, Iba1, and O4), and DAPI on the operated side on postoperative day 7 are shown in Fig 5. Co-localization of GPR37L1 with GFAP was not obvious at low magnification (Fig 5A), but clearly observed at high magnification (Fig 5B). High co-localization of GPR37L1 with Iba1 was frequently observed, even at low magnification (Fig 5C). Co-localization of GPR37L1 with O4 was rare (Fig 5E). Staining with GFAP (Fig 5F) or Iba1 (Fig 5G) revealed significantly more double-positive GPR37L1 cells on the operated side than on the untreated side.

### 3.3 Three-dimensional laser scanning confocal analysis

**Fig 6:**
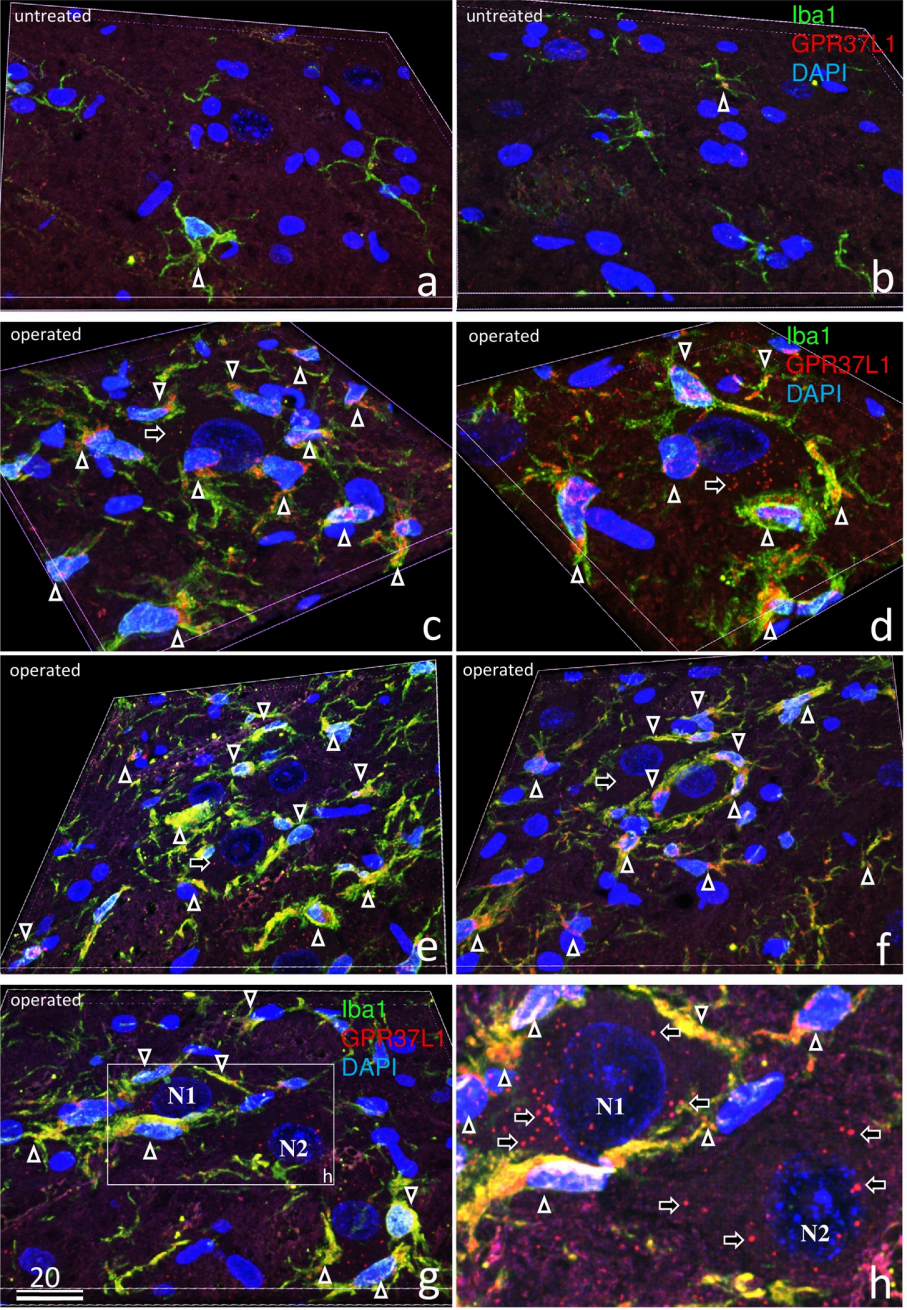
**Three-dimensional laser-scanning confocal analysis of triple-staining with GPR37L1 (red), Iba1 (green), and DAPI (blue) in the untreated (a, b) and operated sides (c–h) of the facial nucleus 7 days after facial nerve transection.** On the untreated side (a, b), there were far fewer microglia than on the operated side, and co-localization of GPR37L1 with Iba1 was rare (arrows in a, b). In contrast, a strong GPR37L1-IR signal (arrows) was observed in the cell bodies of big motoneurons (c, d), small neurons (e, f), and many microglia (c–h). Some microglia gathered around neurons (c), two microglia extended their processes around neurons (d), and some microglia completely covered the neurons (e, f). One neuron (N1 in g, h) was completely covered by many microglia, but the other neighboring neuron (N2 in g, h) was not covered with any microglia. The numbers on the bars indicate the scale in microns.

Three-dimensional laser-scanning confocal analysis of double-staining with GPR37L1, Iba1, and DAPI is shown on the untreated (Fig 6A and 6B) and operated sides (Fig 6C–6H) on postoperative day 7. On the untreated side (Fig 6A and 6B), there were far fewer microglia than on the operated side, and co-localization of GPR37L1 with Iba1 was rare (arrow in Fig 6A). In contrast, GPR37L1-IR was observed in the cell bodies of big motoneurons (Fig 6C and 6D), small neurons (Fig 6E), and microglia around neurons (Fig 6C–6F). In addition, high co-localization of GPR37L1 with Iba1 was clearly shown.

Fig 6C–6F show various stages of microglia covering the neurons: first, activated microglia gather around neurons (Fig 6C); second, they extend their processes around neurons (Fig 6D); and finally, some microglia completely cover the neurons (Fig 6E and 6F). Some neurons (N1 in Fig 6G and 6H) are completely covered by many microglia, whereas other neighboring neurons (N2 in Fig 6G and 6H) are not covered with any.

**Fig 7:**
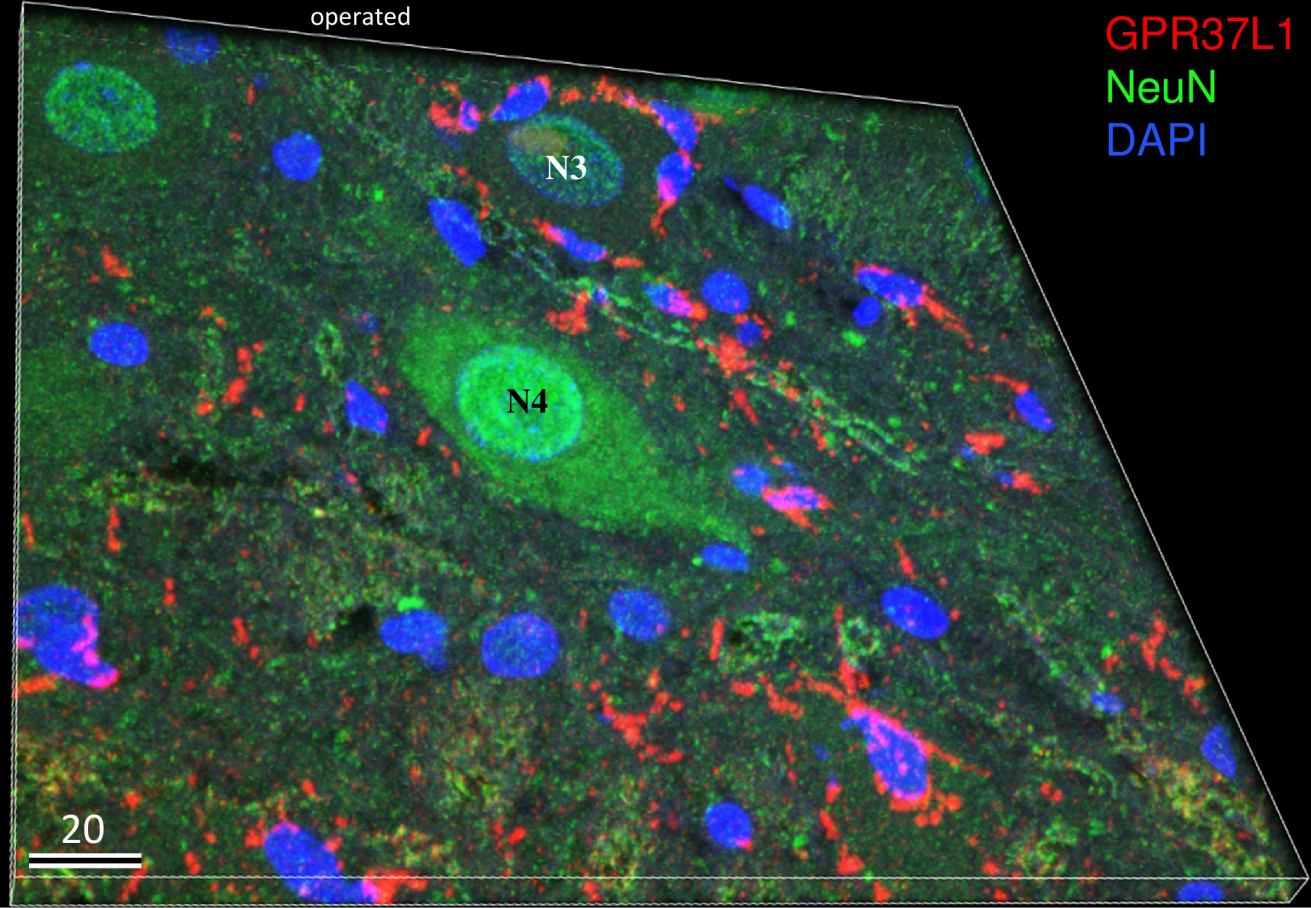
Three-dimensional image of triple-staining with GPR37L1 (red), NeuN (green), and DAPI (blue) on the operated side 7 days after facial nerve transection. GPR37L1-IR was observed in the cell bodies of microglia-like small cells. GPR37L1 co-localization with NeuN was not as prominent in the neurons as in the small cells around the neurons. Note that the neurons in the center of figure with a strong NeuN-IR signal were not surrounded by GPR37L1-IR cells, but the neuron in the upper side of the figure with a faint NeuN-IR signal was surrounded by small GPR37L1-IR cells. The numbers on the bars indicate the scale in microns.

Three-dimensional imaging of double-staining with GPR37L1, NeuN, and DAPI on the operated side on postoperative day 7 is shown in Fig 7. GPR37L1-IR was observed in the cell bodies of many microglia. GPR37L1-IR in NeuN-positive neurons was weak compared with that in microglia around neurons. Notably, the neurons (Fig 7, N4) in the center of the figure with a strong NeuN-IR were not surrounded by GPR37L1-IR cells, but the damaged neurons (Fig 7, N3) with a faint NeuN-IR were surrounded by many GPR37L1-IR microglia-like cells (Fig 7).

## 4 Discussion

### PS has neuroprotective and glioprotective effects

PS plays multifunctional roles both intracellularly as a regulator of lysosomal enzyme activity and extracellularly as a secreted factor with neuroprotective and glioprotective effects. The ability of secreted PS to promote protective effects in the nervous system is known to involve the activation of G proteins, and the orphan GPCRs GPR37 and GPR37L1 have recently been shown to mediate signaling induced by both PS and PS-derived peptides [19,35].

Following facial nerve resection, GPR37-IR was more intense in the cytoplasm of motoneurons on the operated side than on the untreated side (Fig 3F and 3G). In addition, GPR37-IR was markedly higher in microglia (Figs 4H and 5G) and moderately higher in astrocytes (Figs 4D and 5F) on the operated side. Both microglia and astrocytes were 10-fold more numerous on the operated side than on the untreated side (Fig 4D and 4H); however, GPR37L1/Iba1 double-positive cells were much more numerous than GPR37L1/GFAP double-positive cells (Fig 5F and 5G). Using the same facial nerve transection model, previous studies have reported higher PS levels [26], higher PS mRNA expression levels, and higher numbers of PS-positive microglia [27]. Several recent studies have reported that GPR37L1 was mainly present in astrocytes [22,23]. This inconsistency may be due to the animal models used; we applied a nerve transection model, whereas others have applied cultured cells or ischemic models.

### The role of two PS receptors, GPR37 and GPR37L1

GPR37, also known as parkin-associated endothelin-like receptor (Pael-R), is an orphan GPCR that exhibits poor plasma membrane expression when expressed in most cell types. Entire or sequential N-terminal truncations increase the level of surface expression. In studies examining the effects of co-expression of GPR37 with a variety of other GPCRs, the level of GPR37 surface expression was significantly increased [36].

In the present study, GPR37-IR was observed in the neurons, and GPR37-IR in neurons on the operated side was similar to that in neurons on the untreated side. GPR37 surface expression was poor, and overall GPR37 expression was more prominent in the cytoplasm than on the cell surface at days 1, 3, and 7 after neuronal damage, which is typical of most cell types (Fig 2F). Surface expression of GPR37 may be observed in the early stages after nerve transection.

**Fig 8:**
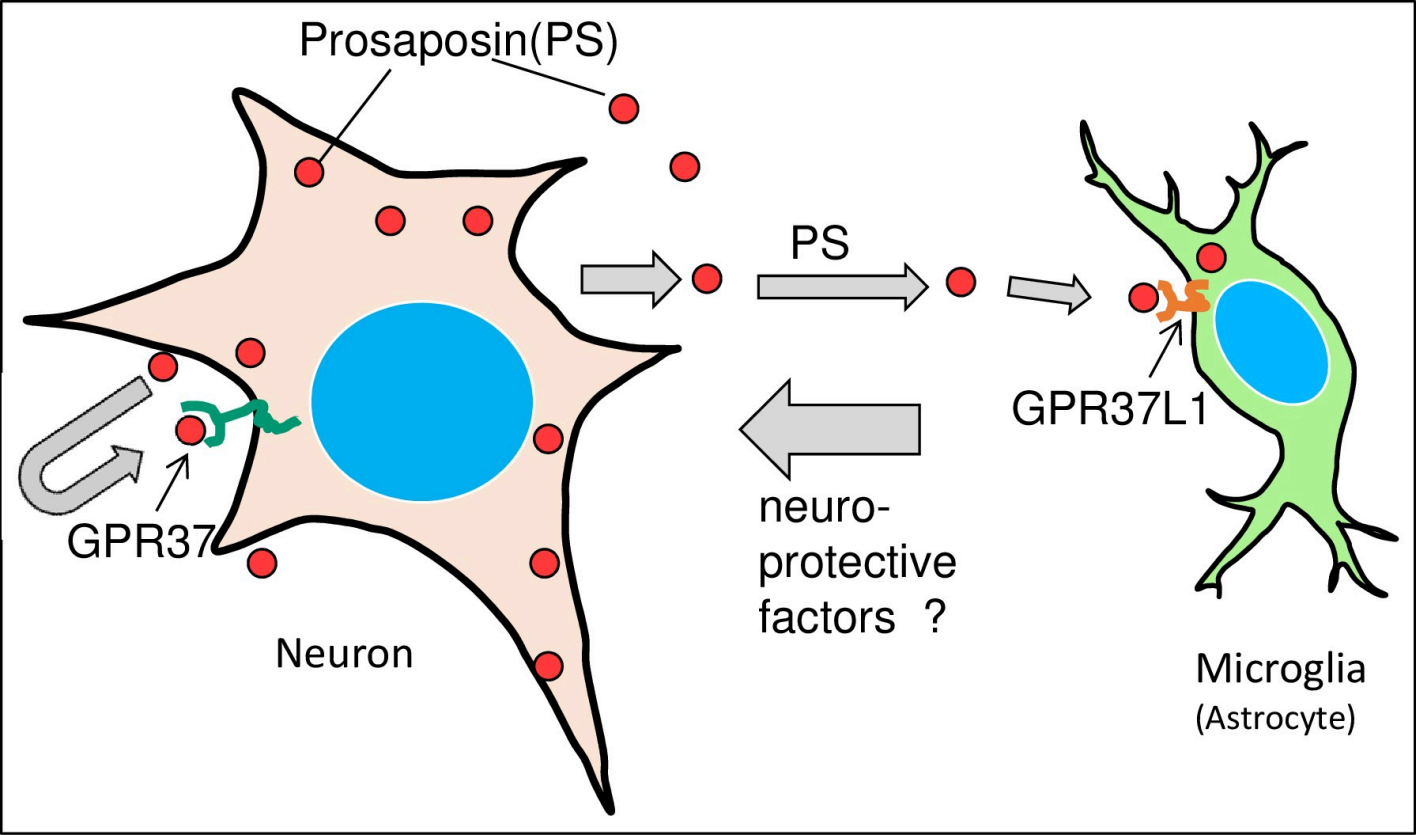
Schematic drawing of the pathological events between neurons and microglia/astrocyte in the facial nucleus after nerve transection. Neuronal damage produces PS, and PS stimulates neuroprotective effects via GPR37. At the same time, secreted PS stimulates microglia or astrocyte via GPR37L1 to produce some neuroprotective factors.

In contrast, GPR37L1-IR was observed in the microglia and astrocyte, and it increased predominantly on the operated side. The number of microglia and astrocyte increased by approximately ten-fold (Fig 4D and 4H). Moreover, microglia with a strong GPR37L1-IR signal covered the damaged neurons (Figs 6C–6H and 7). These pathological events between neurons and microglia in the facial nucleus after nerve transection is summarized in Fig 8. After cellular damage occurs, PS is produced, which stimulates nerve growth via GPR37. At the same time, secreted PS stimulates microglia or astrocyte via GPR37L1 to produce neuroprotective factors to protect the damaged neurons.

Two PS receptors, GPR37 and GPR37L1, showed different distribution patterns. This indicates that these two receptors function independently, at least in damaged nervous tissue. Meyers [19] supports this idea, stating “The difference in findings between the ERK phosphorylation experiments and the oxidative stress protection studies suggests that GPR37 and GPR37L1 do not couple to exactly the same set of intracellular signaling pathways and that the protective actions of prosaptide and PS via GPR37/GPR37L1 are dependent on more than just downstream activation of ERK. Future studies will illuminate the differences between GPR37 and GPR37L1, providing insights into why there seem to be at least two distinct receptors for PS. We have observed that PS in the rat testis was distributed mainly on the basal side of the seminiferous tubules, and the two PS receptors showed roughly complementary distributions: GPR37L1 was strongly distributed in germ cells in the earlier stages of spermatogenesis, whereas GPR37 was present in the later stages [25].

### PS and microglia

PS and PS-derived peptides have been shown to exert effects on Schwann cells [17,18,37], oligodendrocytes [17], astrocytes [19], and some neurons. Few studies, however, have focused on the effects of these peptides on microglia. We reported the important roles of both neurons and microglia during neuroregeneration after facial nerve transection [27]. In that study, *in situ* hybridization signals from PS were observed in both the large neuronal cells and small glial cells, and the number of glial cells on the operated side increased in comparison to that on the untreated side. The number of Iba1-positive cells also increased on the operated side [27]. These results indicate PS production by microglia.

Sekiya et al. [38] demonstrated a marked decrease in microglial cell numbers in carbon monoxide (CO)-exposed brain tissues, but not in hypoxic brains. They found that 1) demyelination induced by CO was more severe and persisted longer than that induced by pure hypoxia, 2) microglial cells decreased after CO exposure, and 3) neurotrophic factors decreased after CO exposure compared with levels after low O_2_ exposure. The authors suspected causal involvement of the decreased neurotrophic actions by microglia in the degeneration of neurons, oligodendrocyte progenitor cells, and oligodendrocytes [38]. Therefore, protection of microglia from CO-induced damage or supplementation with neurotrophic factors such as PS or related peptides may become a promising strategy for the treatment or prevention of CO-induced delayed encephalopathy.

## Supporting information

S1 Fig Original figures of Fig 5. (PPTX)

S2 Fig Original figure of the facial nerve of Fig 1. (TIF)

S3 Fig Original figures of Fig 2. (ZIP)

S4 Fig Original figures of Fig 3. (ZIP)

S5 Fig Original figures of Fig 4. (ZIP)

S6 Fig Original figures of Fig 6. (ZIP)

S7 Fig Original figure of Fig 7. (TIF)

S1 Dataset Supporting information of S2 Fig. (XLSX)

S1 Text (PDF)
