# Supplementary material for: Prosaposin and its receptors GRP37 and GPR37L1 show increased immunoreactivity in the facial nucleus following facial nerve transection
Source: PLoS One. 2020 Dec 1;15(12):e0241315. doi: 10.1371/journal.pone.0241315 (PMC7707515; doi:10.1371/journal.pone.0241315)
Supplement: S1 Fig — (PPTX) [file pone.0241315.s001.pptx]

## Slide 1
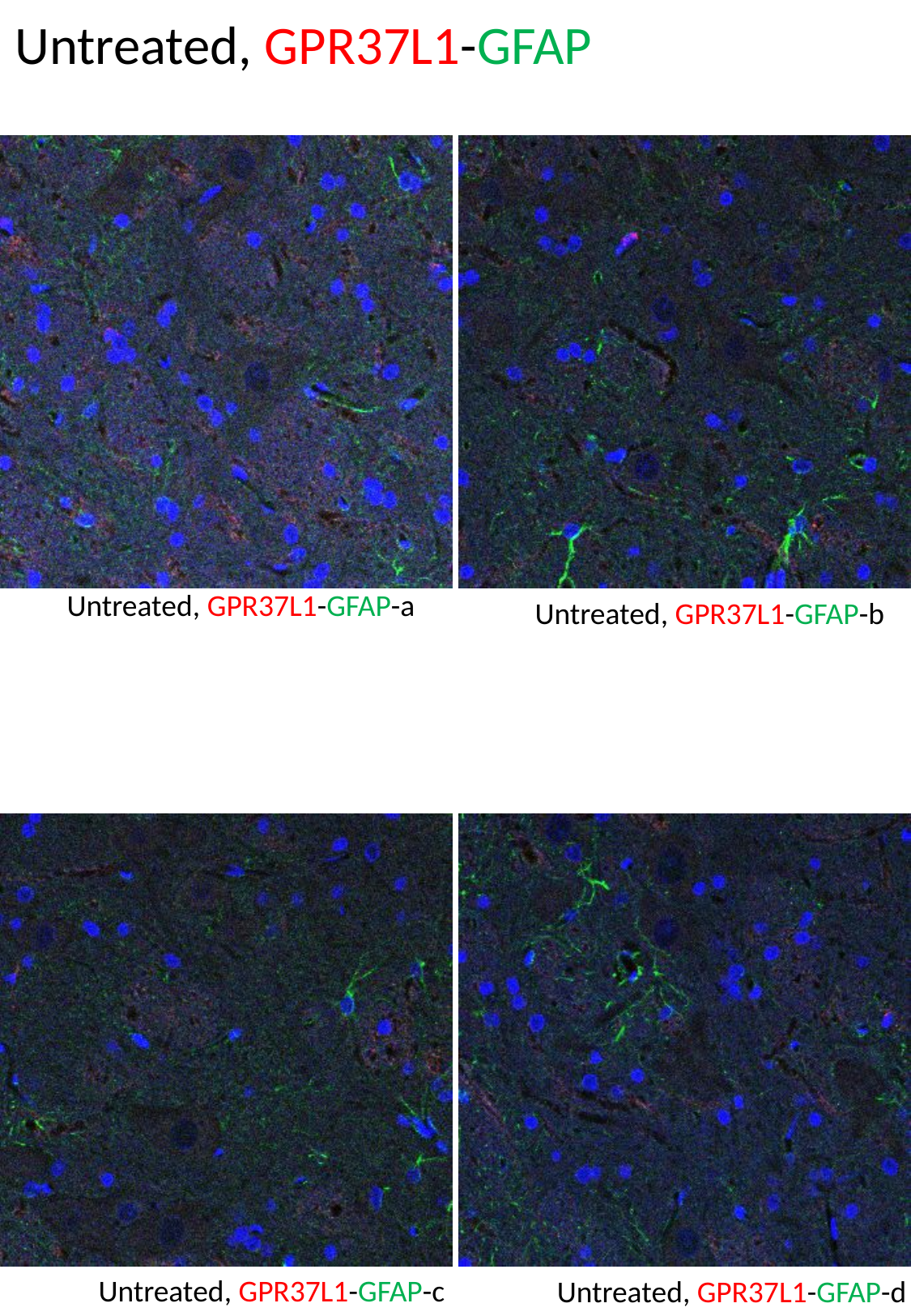

Untreated, GPR37L1-GFAP
Untreated, GPR37L1-GFAP-a
Untreated, GPR37L1-GFAP-b
Untreated, GPR37L1-GFAP-c
Untreated, GPR37L1-GFAP-d

## Slide 2
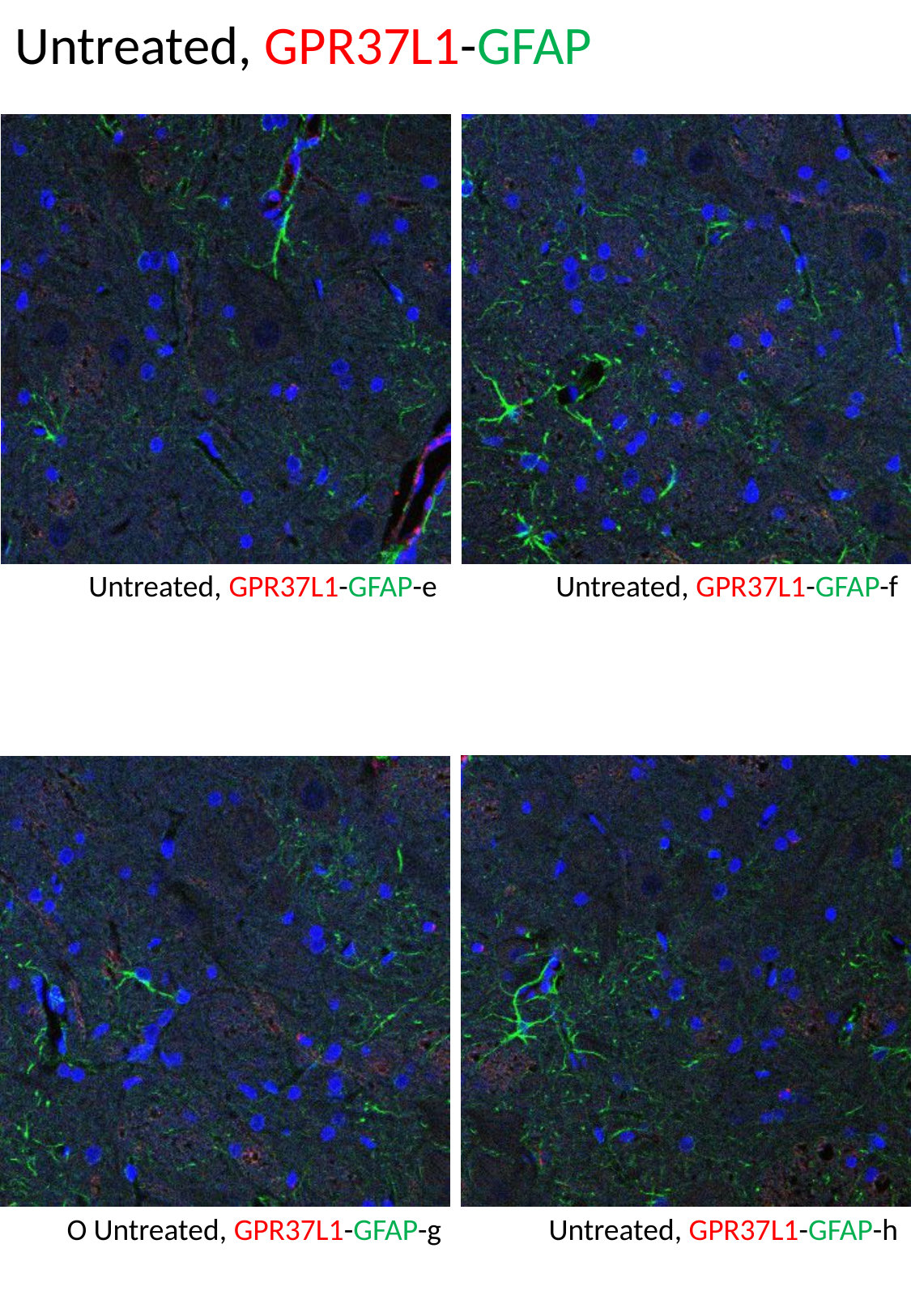

Untreated, GPR37L1-GFAP
Untreated, GPR37L1-GFAP-f
Untreated, GPR37L1-GFAP-e
O Untreated, GPR37L1-GFAP-g
Untreated, GPR37L1-GFAP-h

## Slide 3
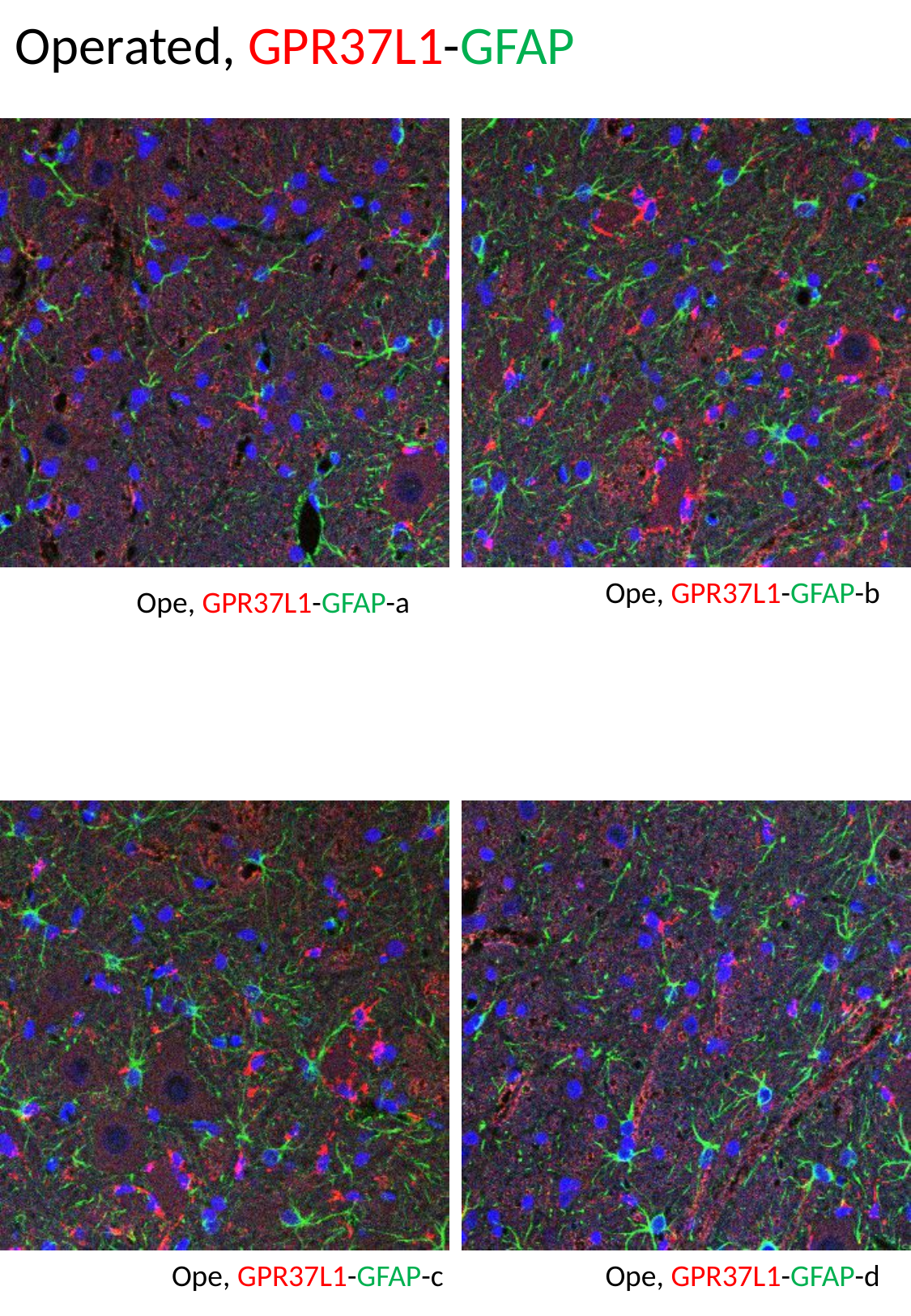

Operated, GPR37L1-GFAP
Ope, GPR37L1-GFAP-b
Ope, GPR37L1-GFAP-a
Ope, GPR37L1-GFAP-c
Ope, GPR37L1-GFAP-d

## Slide 4
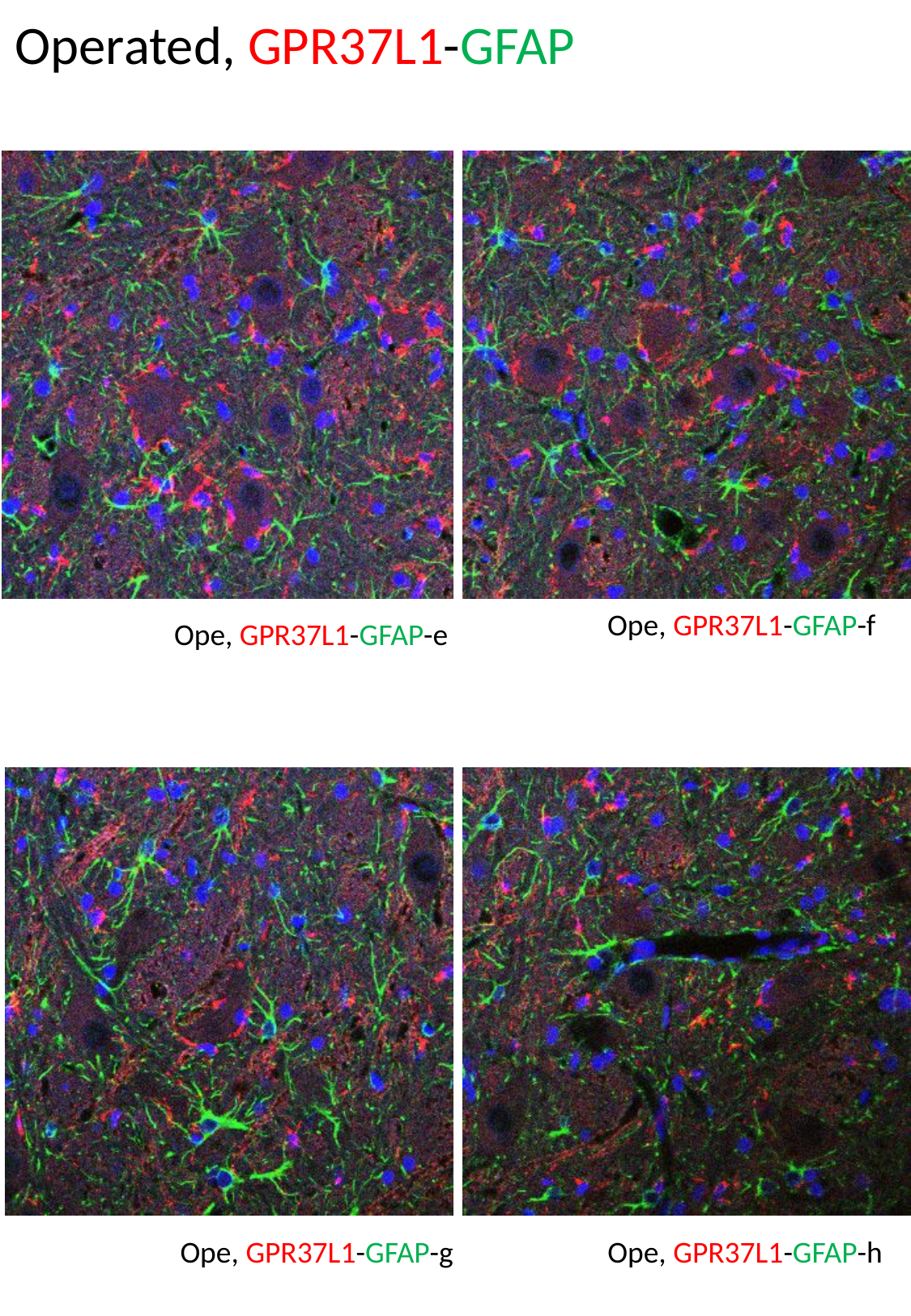

Operated, GPR37L1-GFAP
Ope, GPR37L1-GFAP-f
Ope, GPR37L1-GFAP-e
Ope, GPR37L1-GFAP-g
Ope, GPR37L1-GFAP-h

## Slide 5
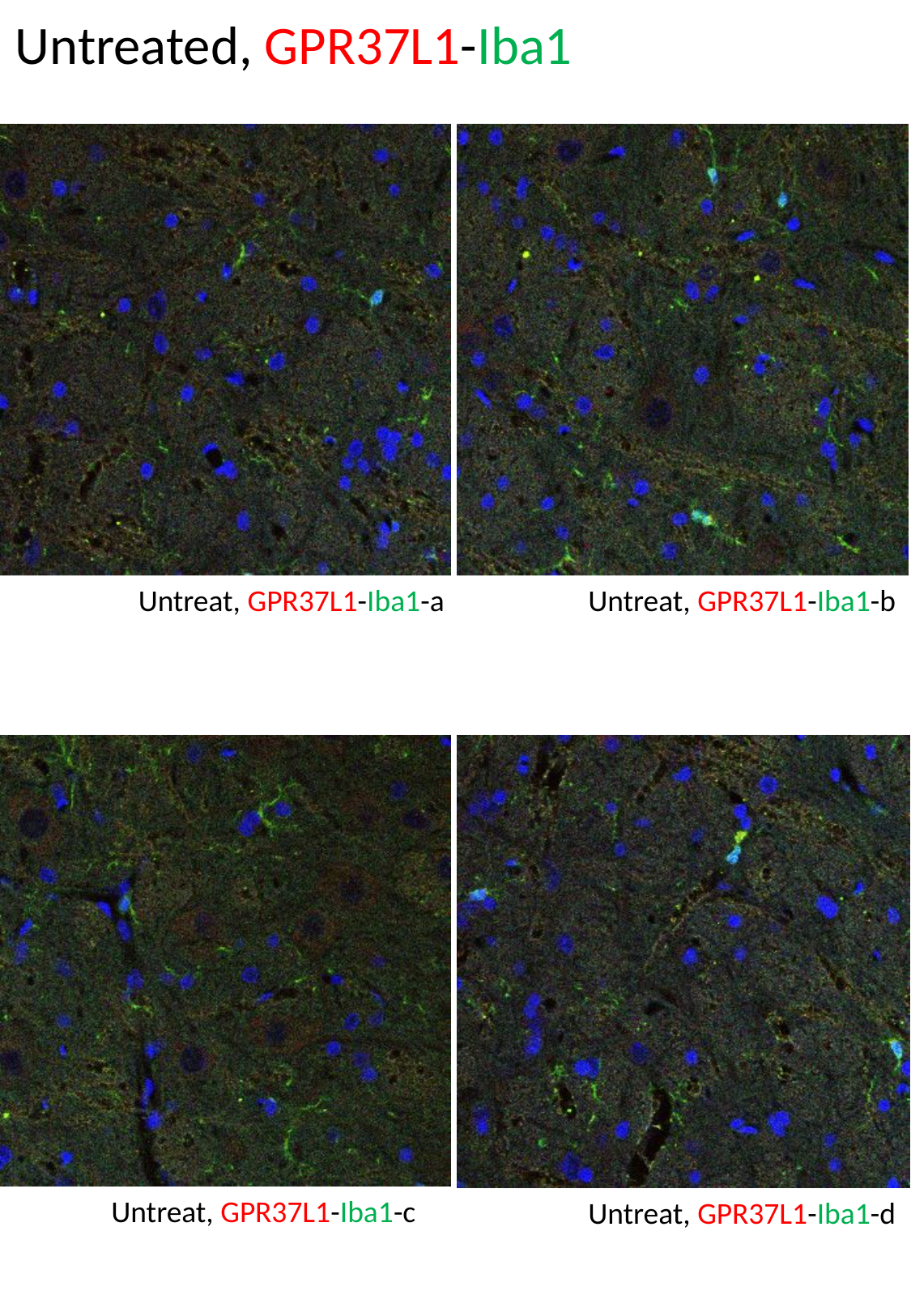

Untreated, GPR37L1-Iba1
Untreat, GPR37L1-Iba1-a
Untreat, GPR37L1-Iba1-b
Untreat, GPR37L1-Iba1-c
Untreat, GPR37L1-Iba1-d

## Slide 6
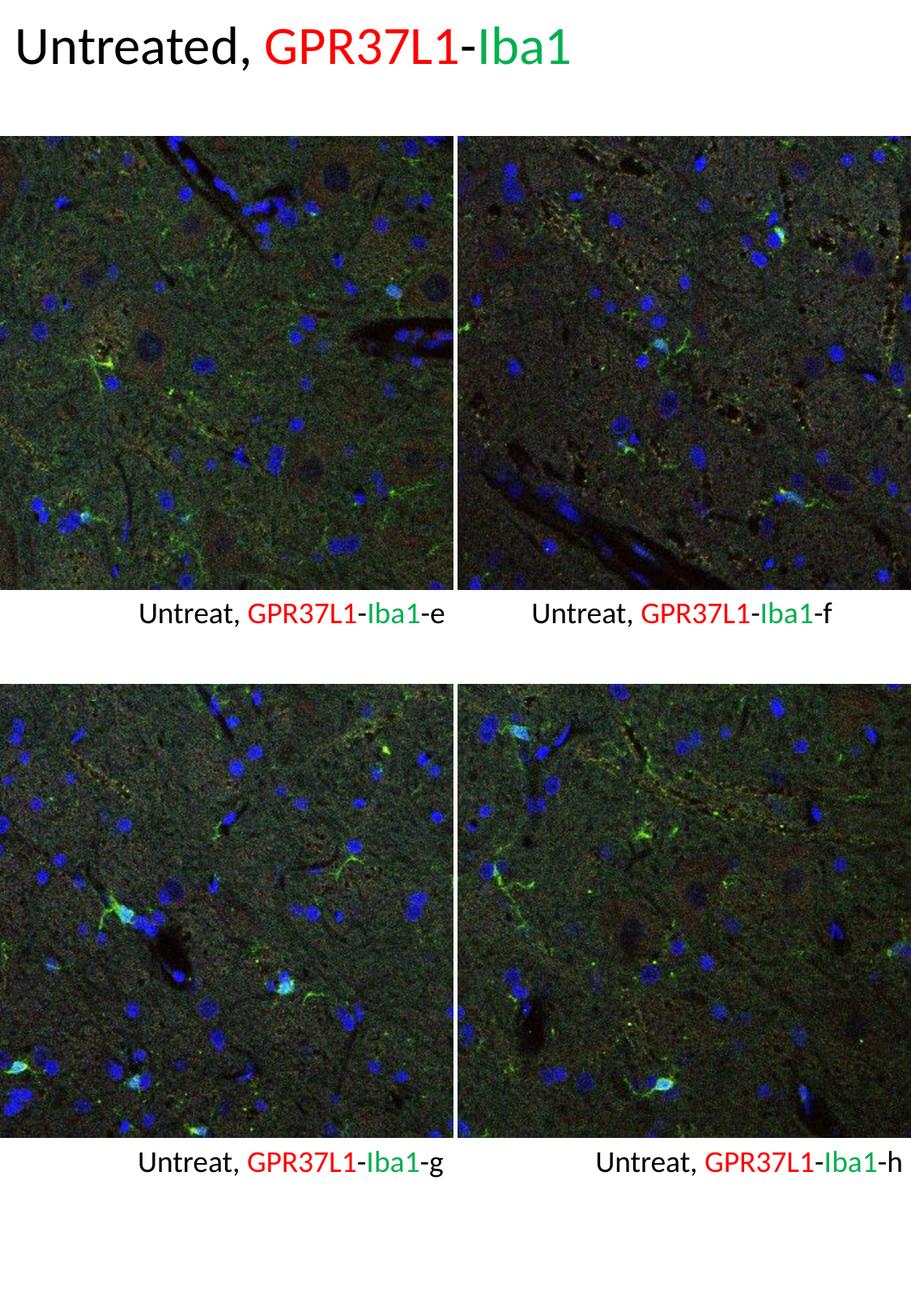

Untreated, GPR37L1-Iba1
Untreat, GPR37L1-Iba1-f
Untreat, GPR37L1-Iba1-e
Untreat, GPR37L1-Iba1-g
Untreat, GPR37L1-Iba1-h

## Slide 7
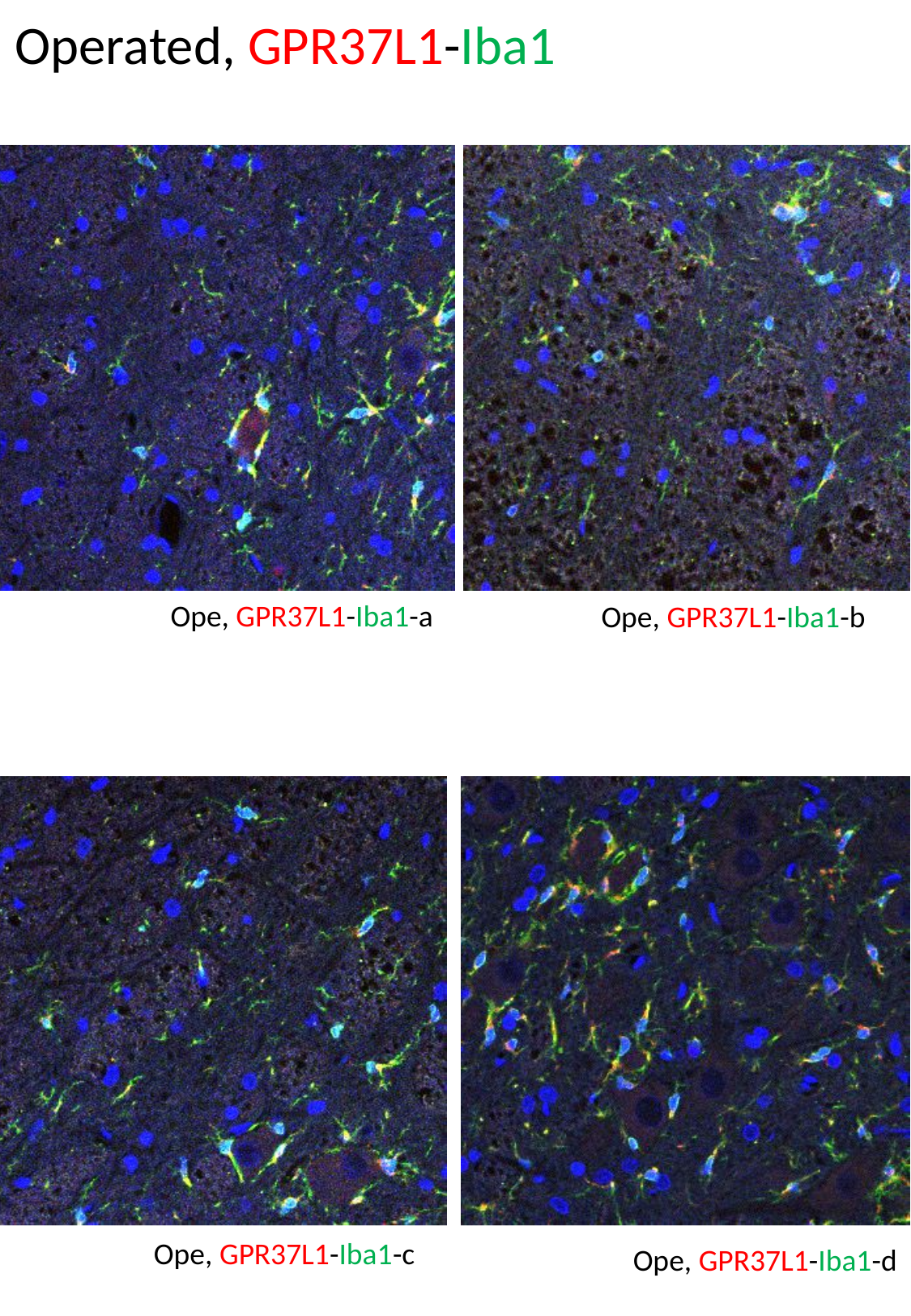

Operated, GPR37L1-Iba1
Ope, GPR37L1-Iba1-a
Ope, GPR37L1-Iba1-b
Ope, GPR37L1-Iba1-c
Ope, GPR37L1-Iba1-d

## Slide 8
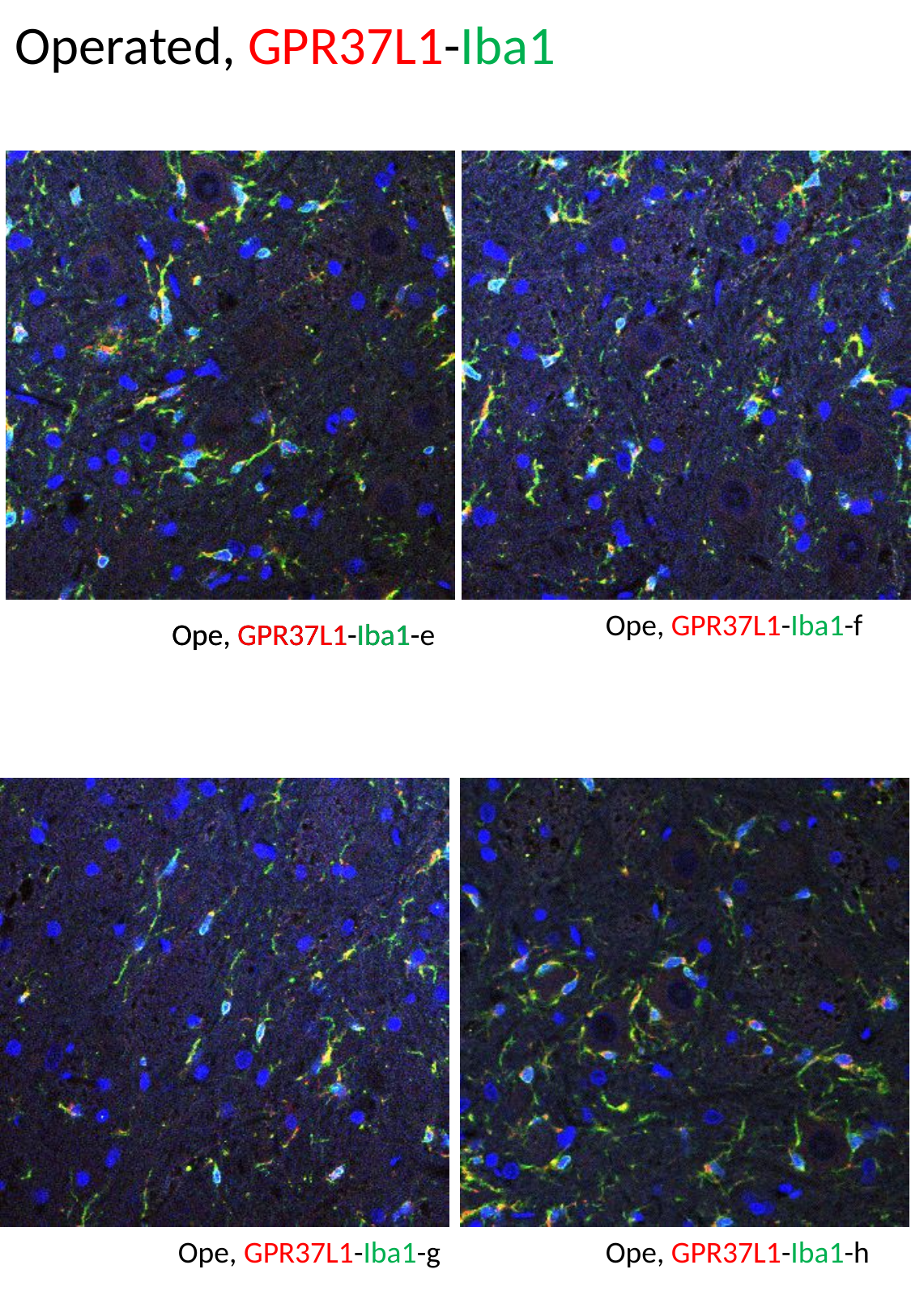

Operated, GPR37L1-Iba1
Ope, GPR37L1-Iba1-f
Ope, GPR37L1-Iba1-e
Ope, GPR37L1-Iba1-
Ope, GPR37L1-Iba1-g
Ope, GPR37L1-Iba1-h

## Slide 9
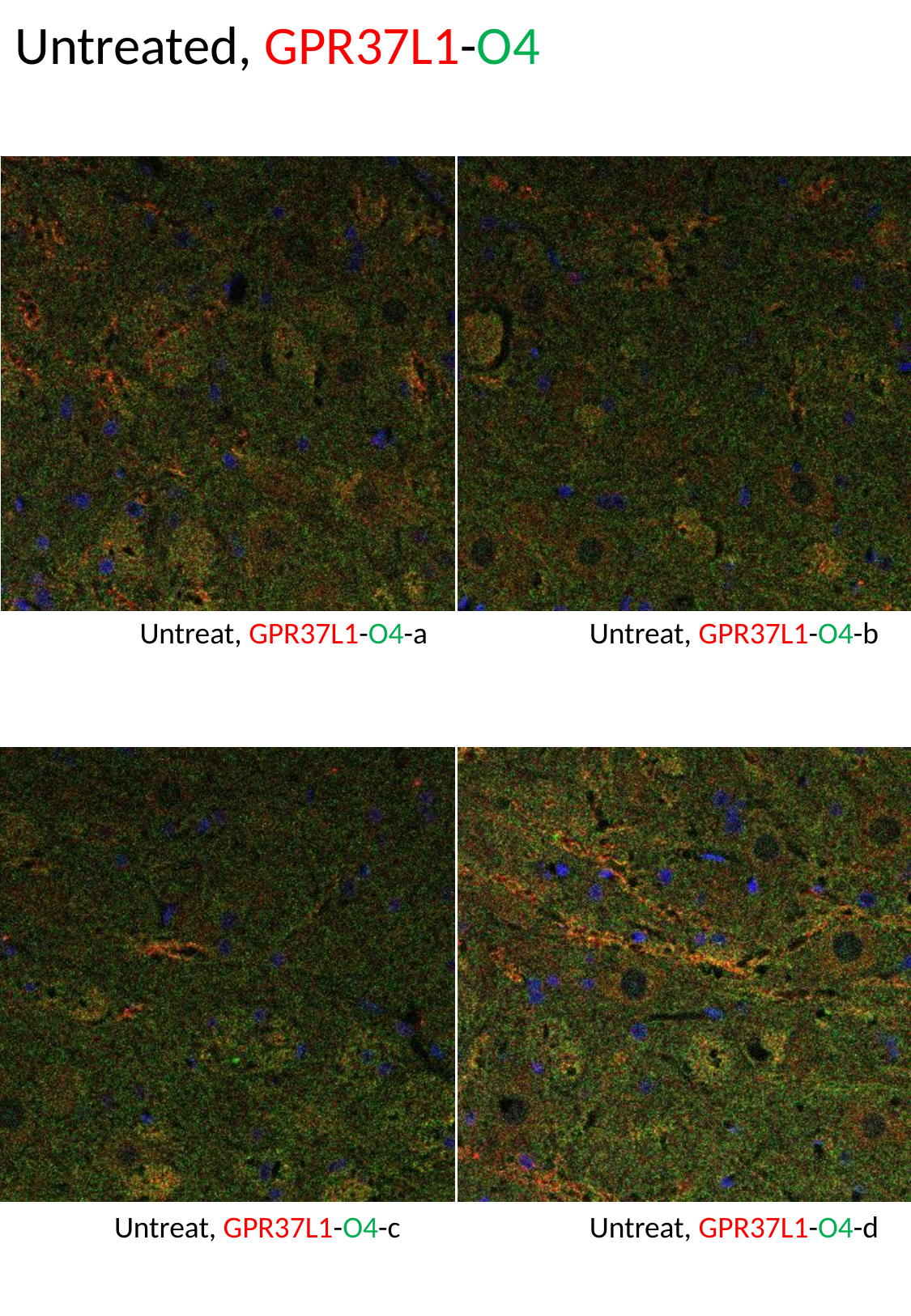

Untreated, GPR37L1-O4
Untreat, GPR37L1-O4-a
Untreat, GPR37L1-O4-b
Untreat, GPR37L1-O4-c
Untreat, GPR37L1-O4-d

## Slide 10
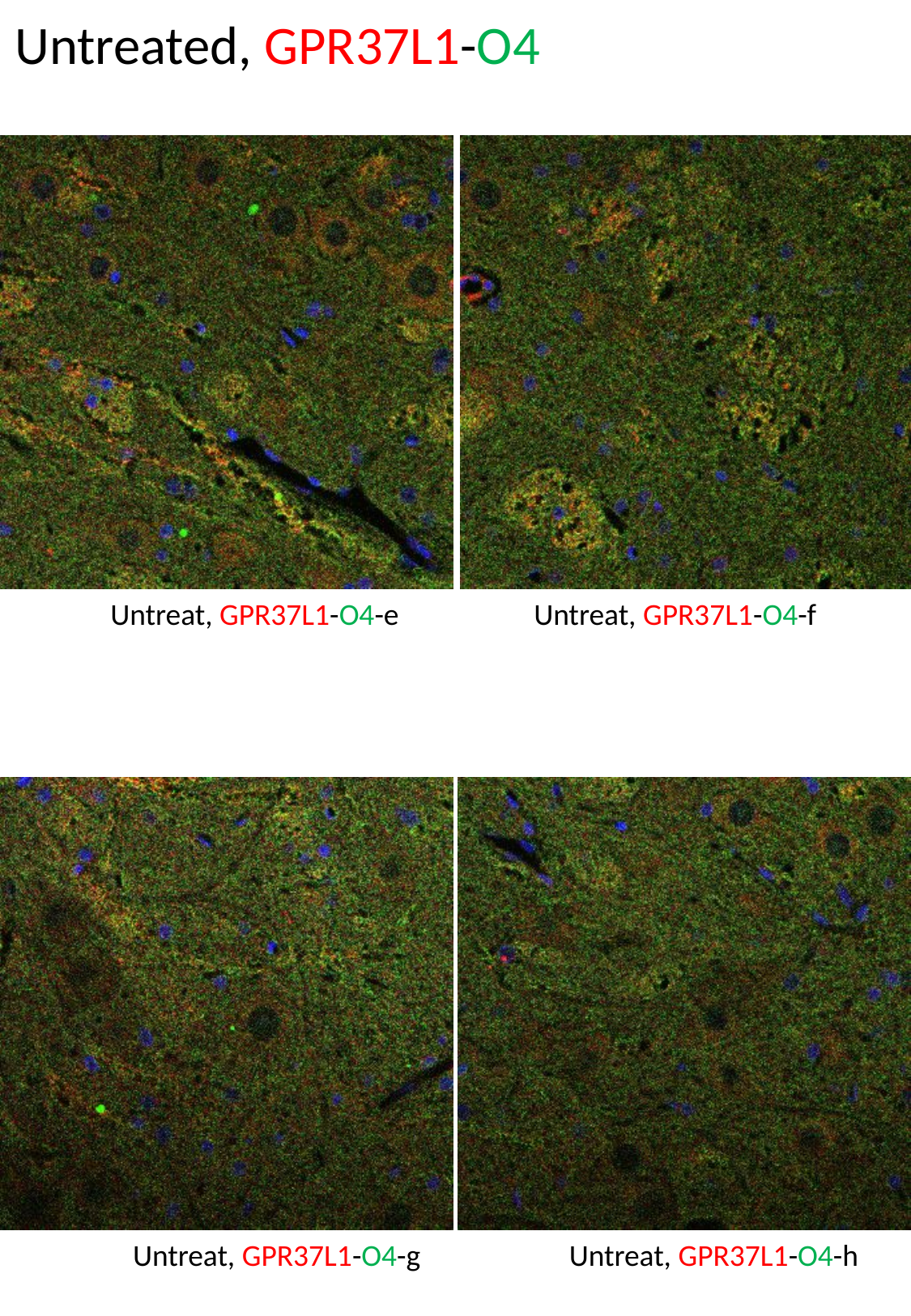

Untreated, GPR37L1-O4
Untreat, GPR37L1-O4-e
Untreat, GPR37L1-O4-f
Untreat, GPR37L1-O4-g
Untreat, GPR37L1-O4-h

## Slide 11
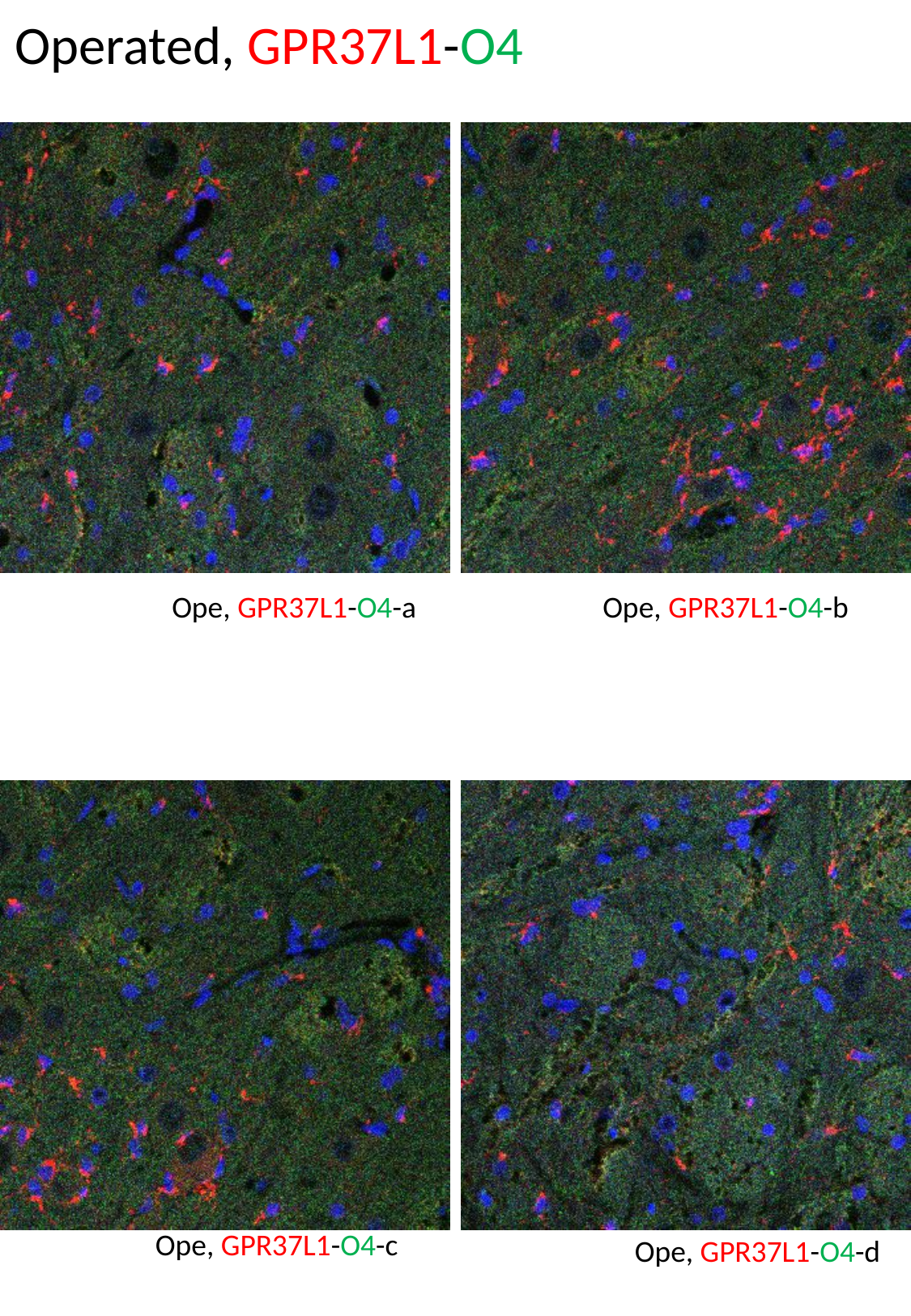

Operated, GPR37L1-O4
Ope, GPR37L1-O4-a
Ope, GPR37L1-O4-b
Ope, GPR37L1-O4-c
Ope, GPR37L1-O4-d

## Slide 12
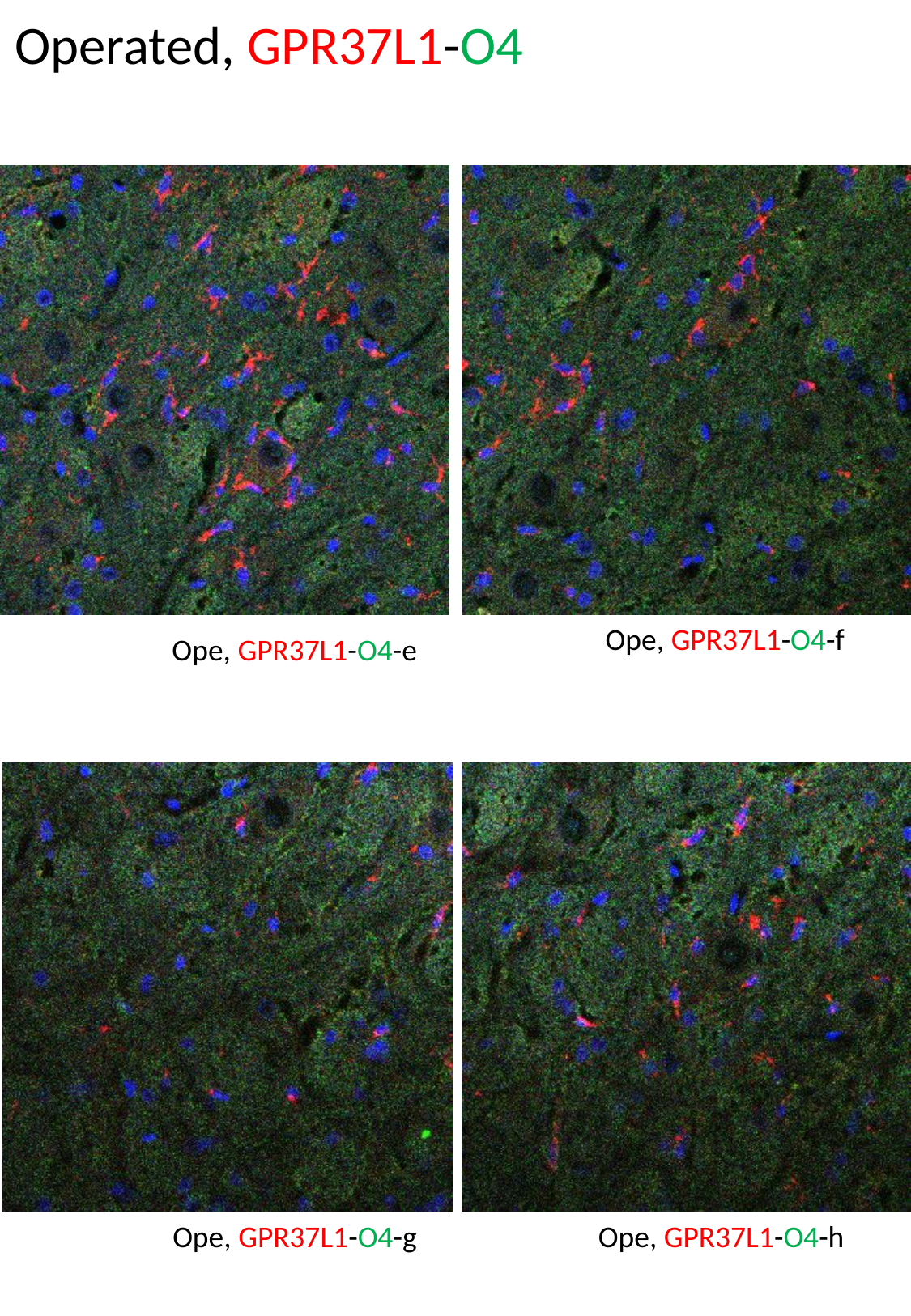

Operated, GPR37L1-O4
Ope, GPR37L1-O4-f
Ope, GPR37L1-O4-e
Ope, GPR37L1-O4-g
Ope, GPR37L1-O4-h
