# Supplementary figures and images for: Prosaposin and its receptors GRP37 and GPR37L1 show increased immunoreactivity in the facial nucleus following facial nerve transection
Source: PLoS One. 2020 Dec 1;15(12):e0241315. doi: 10.1371/journal.pone.0241315 (PMC7707515; doi:10.1371/journal.pone.0241315)

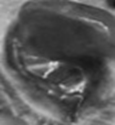

Supplement: S2 Fig — (TIF) [file pone.0241315.s002.tif]

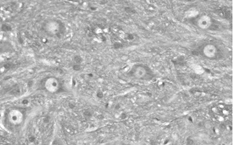

Supplement: S3 Fig — (ZIP) [file pone.0241315.s003.zip › GPR37 ope.tif]

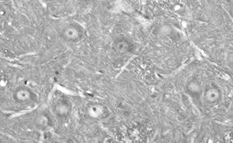

Supplement: S3 Fig — (ZIP) [file pone.0241315.s003.zip › GPR37 unope.tif]

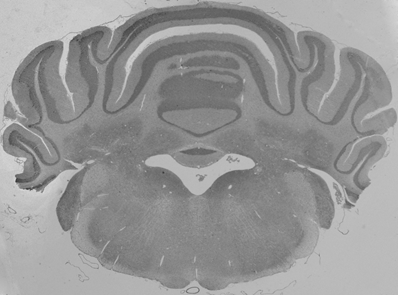

Supplement: S3 Fig — (ZIP) [file pone.0241315.s003.zip › GPR37 whole.tif]

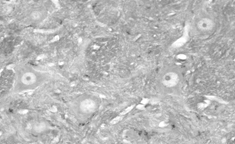

Supplement: S3 Fig — (ZIP) [file pone.0241315.s003.zip › GPR37L1 ope.tif]

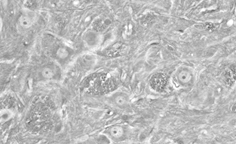

Supplement: S3 Fig — (ZIP) [file pone.0241315.s003.zip › GPR37L1 unope.tif]

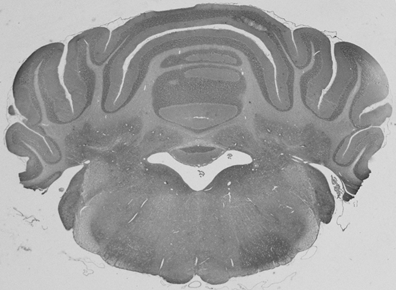

Supplement: S3 Fig — (ZIP) [file pone.0241315.s003.zip › GPR37L1 whole.tif]

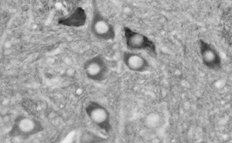

Supplement: S3 Fig — (ZIP) [file pone.0241315.s003.zip › IM1 ope.tif]

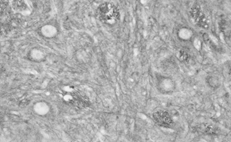

Supplement: S3 Fig — (ZIP) [file pone.0241315.s003.zip › IM1 unope.tif]

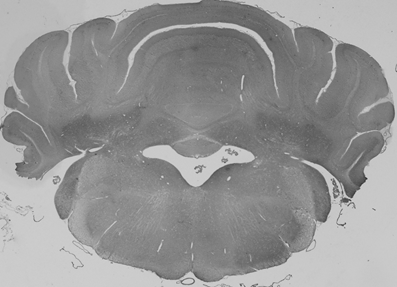

Supplement: S3 Fig — (ZIP) [file pone.0241315.s003.zip › IM1 whole.tif]

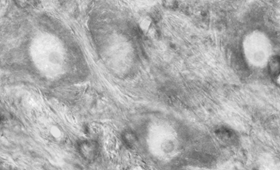

Supplement: S4 Fig — (ZIP) [file pone.0241315.s004.zip › GPR37 1day.tif]

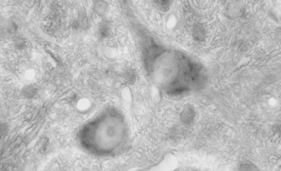

Supplement: S4 Fig — (ZIP) [file pone.0241315.s004.zip › GPR37 3days.tif]

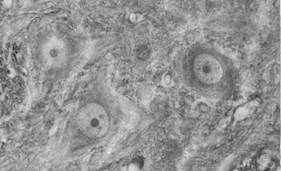

Supplement: S4 Fig — (ZIP) [file pone.0241315.s004.zip › GPR37 7days control.tif]

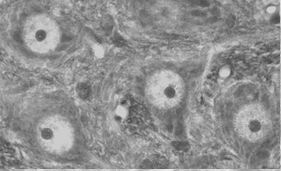

Supplement: S4 Fig — (ZIP) [file pone.0241315.s004.zip › GPR37 7days.tif]

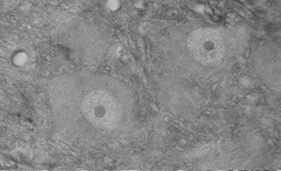

Supplement: S4 Fig — (ZIP) [file pone.0241315.s004.zip › GPR37L1 1day.tif]

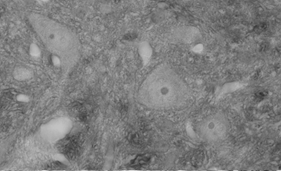

Supplement: S4 Fig — (ZIP) [file pone.0241315.s004.zip › GPR37L1 3days.tif]

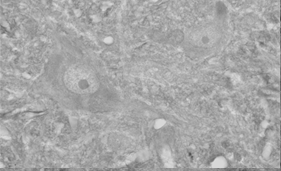

Supplement: S4 Fig — (ZIP) [file pone.0241315.s004.zip › GPR37L1 7days control.png]

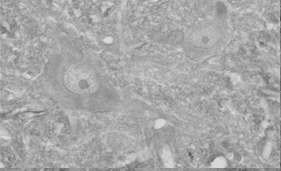

Supplement: S4 Fig — (ZIP) [file pone.0241315.s004.zip › GPR37L1 7days control.tif]

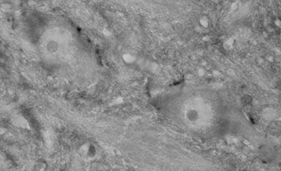

Supplement: S4 Fig — (ZIP) [file pone.0241315.s004.zip › GPR37L1 7days.tif]

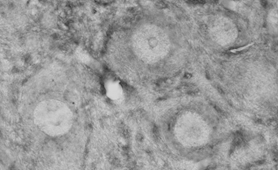

Supplement: S4 Fig — (ZIP) [file pone.0241315.s004.zip › IM1 1day.tif]

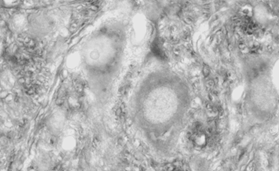

Supplement: S4 Fig — (ZIP) [file pone.0241315.s004.zip › IM1 3days.tif]

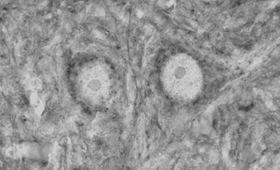

Supplement: S4 Fig — (ZIP) [file pone.0241315.s004.zip › IM1 7days control.tif]

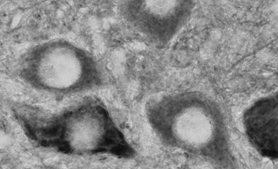

Supplement: S4 Fig — (ZIP) [file pone.0241315.s004.zip › IM1 7days.tif]

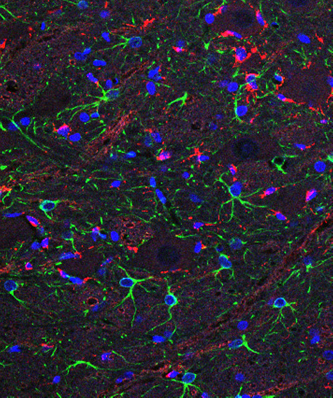

Supplement: S5 Fig — (ZIP) [file pone.0241315.s005.zip › GFAP ope.tif]

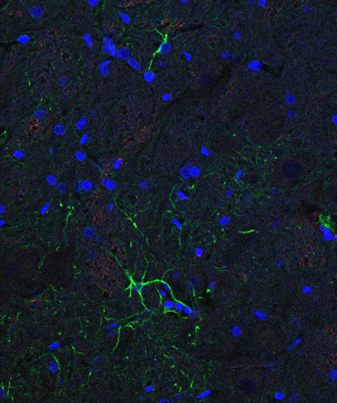

Supplement: S5 Fig — (ZIP) [file pone.0241315.s005.zip › GFAP unope.tif]

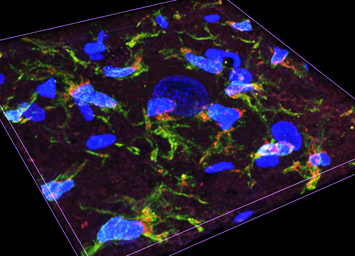

Supplement: S6 Fig — (ZIP) [file pone.0241315.s006.zip › ope c.tif]

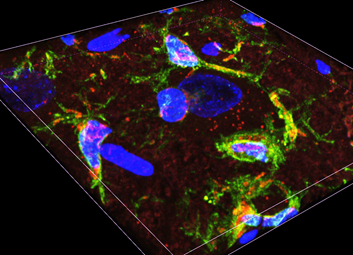

Supplement: S6 Fig — (ZIP) [file pone.0241315.s006.zip › ope d.tif]

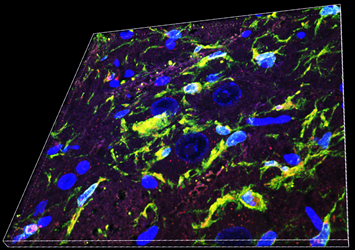

Supplement: S6 Fig — (ZIP) [file pone.0241315.s006.zip › ope e.tif]

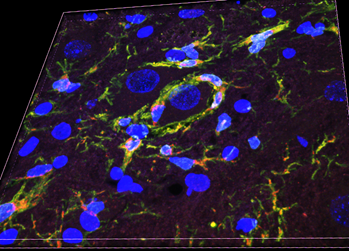

Supplement: S6 Fig — (ZIP) [file pone.0241315.s006.zip › ope f.tif]

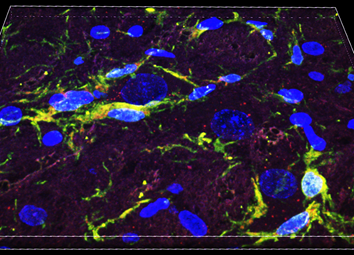

Supplement: S6 Fig — (ZIP) [file pone.0241315.s006.zip › ope g.tif]

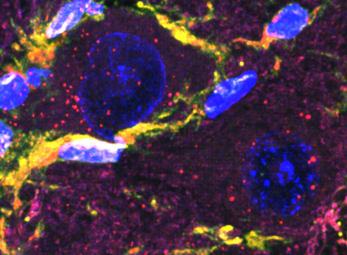

Supplement: S6 Fig — (ZIP) [file pone.0241315.s006.zip › ope g2.tif]

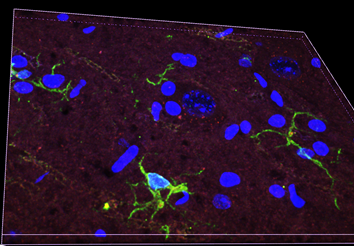

Supplement: S6 Fig — (ZIP) [file pone.0241315.s006.zip › unope a.tif]

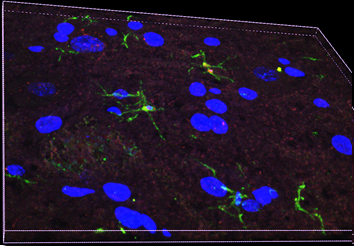

Supplement: S6 Fig — (ZIP) [file pone.0241315.s006.zip › unope b.tif]

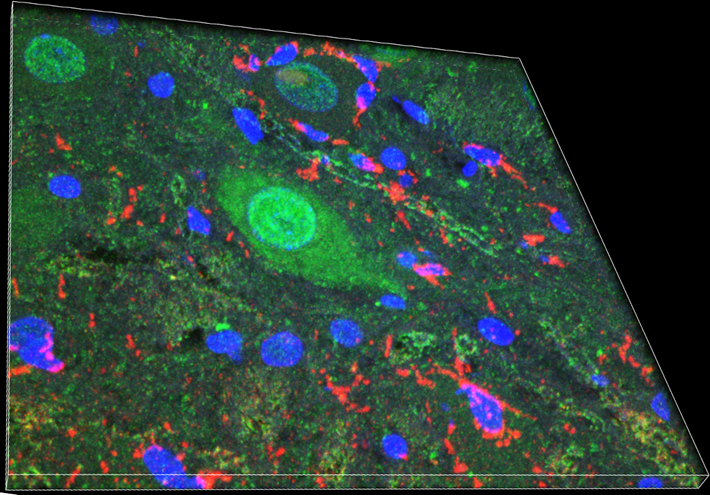

Supplement: S7 Fig — (TIF) [file pone.0241315.s007.tif]
