# Supplementary material for: Prosaposin and its receptors GRP37 and GPR37L1 show increased immunoreactivity in the facial nucleus following facial nerve transection
Source: PLoS One. 2020 Dec 1;15(12):e0241315. doi: 10.1371/journal.pone.0241315 (PMC7707515; doi:10.1371/journal.pone.0241315)
Supplement: S1 Text — (PDF) [file pone.0241315.s009.pdf]

Textcheck

www.textcheck.com

Textcheck Certificate

Refnum:	19060802
Title:	Prosaposin receptors in the facial nucleus after facial nerve transection
Date:	2019/06/11

We hereby certify that Textcheck has checked and corrected the English in the manuscript named above.

A specialist editor with suitable professional knowledge (M.Sc. or Ph.D./M.D.) reviewed and corrected the English. An English language specialist subsequently checked the paper again. The first language of both editors is English.

Please direct any questions regarding this certificate or the English in the certified paper to: certified@textcheck.com (Please quote our reference number: '19060802')
